# Harnessing the runoff reduction potential of urban bioswales as an adaptation response to climate change

**DOI:** 10.1038/s41598-024-61878-7

**Published:** 2024-05-28

**Authors:** Lingwen Lu, Matthew Johnson, Fangfang Zhu, Yaoyang Xu, Tian Ruan, Faith Ka Shun Chan

**Affiliations:** 1 School of Geographical Sciences, Faculty of Science and Engineering, University of Nottingham Ningbo China, Ningbo, 315100 China; 2 Key Laboratory of Urban Environment and Health, Ningbo Observation and Research Station, Institute of Urban Environment, Chinese Academy of Sciences (CAS), Xiamen, 361021 China; 3 School of Geography, University of Nottingham, Nottingham, Nottinghamshire NG7 2RD UK; 4 Department of Civil Engineering, Faculty of Science and Engineering, University of Nottingham Ningbo China, Ningbo, 315100 China; 5 Zhejiang Key Laboratory of Urban Environmental Processes and Pollution Control, CAS Haixi Industrial Technology Innovation Centre in Beilun, Ningbo, 315830 China; 6 Water@Leeds Research Institute, University of Leeds, Leeds, LS2 9JT UK

**Keywords:** Environmental sciences, Climate change, Hydrology

## Abstract

Nature-based solutions (NbS), including China's Sponge City Program (SCP), can address the challenges urban communities face due to surface runoff and flooding. The current capacity of SCP facilities in urban environments falls short of meeting the demands placed on communities by climate change. Bioswales are a form of SCP facility that plays an important role in reducing surface runoff by promoting infiltration. This study assesses the potential of SCP facilities to reduce runoff in urban communities under climate change using the storm water management model. The study site in Ningbo, China, was used to evaluate the potential role of bioswales in reducing runoff risks from climate change. We found that bioswales were most effective in scenarios when rainfall peaks occurred early and were less effective in right-skewed rainfall events. The overall performance of SCP facilities was similar across all climate scenarios. To maintain the current protection level of SCP facilities, bioswales would need to cover at least 4% of the catchment area. These findings from Ningbo provide a useful method for assessing NbS in other regions and indicative values for the increase in the bioswale coverage needed to adapt to climate change.

## Introduction

Urban populations are rapidly increasing, with 6.7 billion people (68.4% of the global population) projected to live in cities by 2050, compared to 4.4 billion today^1^. Anthropogenic changes to, and control of, hydrological processes in urban areas, coupled with ongoing climate change enhance global urban populations being increasingly at risk of flooding^2^ and water scarcity^3^, sometimes in the same city at different times of year. Traditional drainage systems have left urban infrastructure vulnerable to flooding, particularly due to the increased frequency and intensity of extreme precipitation brought on by climate change. More urbanised communities with impermeable areas increased the flooding risks to people. For example, approximately 1.81 billion people (23% of the global population) are currently exposed to 1-in-100-year return period flooding^4^. As such, a paradigm shift in the management of urban water is necessary to enhance the societal and ecosystem services urban freshwaters provide whilst reducing hazards^5–7^. This shift is currently taking place, with a move away from grey infrastructure and piped drainage, and towards nature-based solutions (NbS) that work alongside and alleviate pressure on grey infrastructure, promoting more natural hydrological processes within urban areas, as emphasised by the 2022 report from the Intergovernmental Panel on Climate Change (IPCC) Working Group II^8^.

NbS is an umbrella concept that encompasses a variety of relevant methods for managing stormwater, including best management practices (BMP)^9^, low impact development (LID)^10^, green infrastructure (GI)^11^, blue–green infrastructure (BGI)^12^, Sponge City Program (SCP)^13^, sustainable urban drainage systems (SUDS)^14^, and water-sensitive urban design (WSUD)^15^. These methods have been adopted to mitigate the adverse impacts of serious urban waterlogging and environmental problems in the context of climate change and urbanisation. Swales are one of the oldest and most widely used stormwater management strategies, often utilized in urban areas to channel and purify stormwater away from road surfaces worldwide. Green swales cover a variety of designs and implementations, including grass swales, infiltration swales, bioswales and wet swales. Similar characteristics, including being relatively simple structures, with small footprints, and requiring low maintenance, but also have important differences related to their infiltration capacity and length of time they remain wet (see Table S1 and Fig. S1 in the Supplementary material). They have an important role in combating waterlogging and environmental risks worldwide.

Studies have investigated the effectiveness of green swales in controlling stormwater quality and quantity under various conditions. In a field study of 52 consecutive storm events close to a highway in Maryland, USA, over 4.5 years, it was discovered that grass swales significantly reduced the total volume and velocity of run-off where rainfall was less than 30 mm^16^. Swales did not decrease the volume of runoff during larger events but instead equalized fluctuations in runoff volumes during the event duration^16^. That study was based on two swale design schemes: grass swales and infiltration swales. The effectiveness of infiltration in swales was related to swale slope length in Minnesota, USA^17^. In a recent large-scale experimental investigation conducted in Trondheim City, Norway, it was discovered that infiltration swales (a type of bioswale with a clean homogeneous sand layer for bioretention) reduced runoff to a greater degree than traditional bioswales (soil for bioretention) because of greater drainage efficiency^18^. These studies typically look at only one or two swale types in situ and only consider water quantity, and not the potential for swales to also improve water quality. They also do not compare the performance in terms of water quality and water quantity of more traditional swale types (i.e., grass swales) and more novel swale designs such as bioswales under different rainfall patterns. Overall, there is a relative lack of studies on the recently emerging field of bioswales (Table S1) and there is little systematic research or best-practice advice.

**Figure 1:**
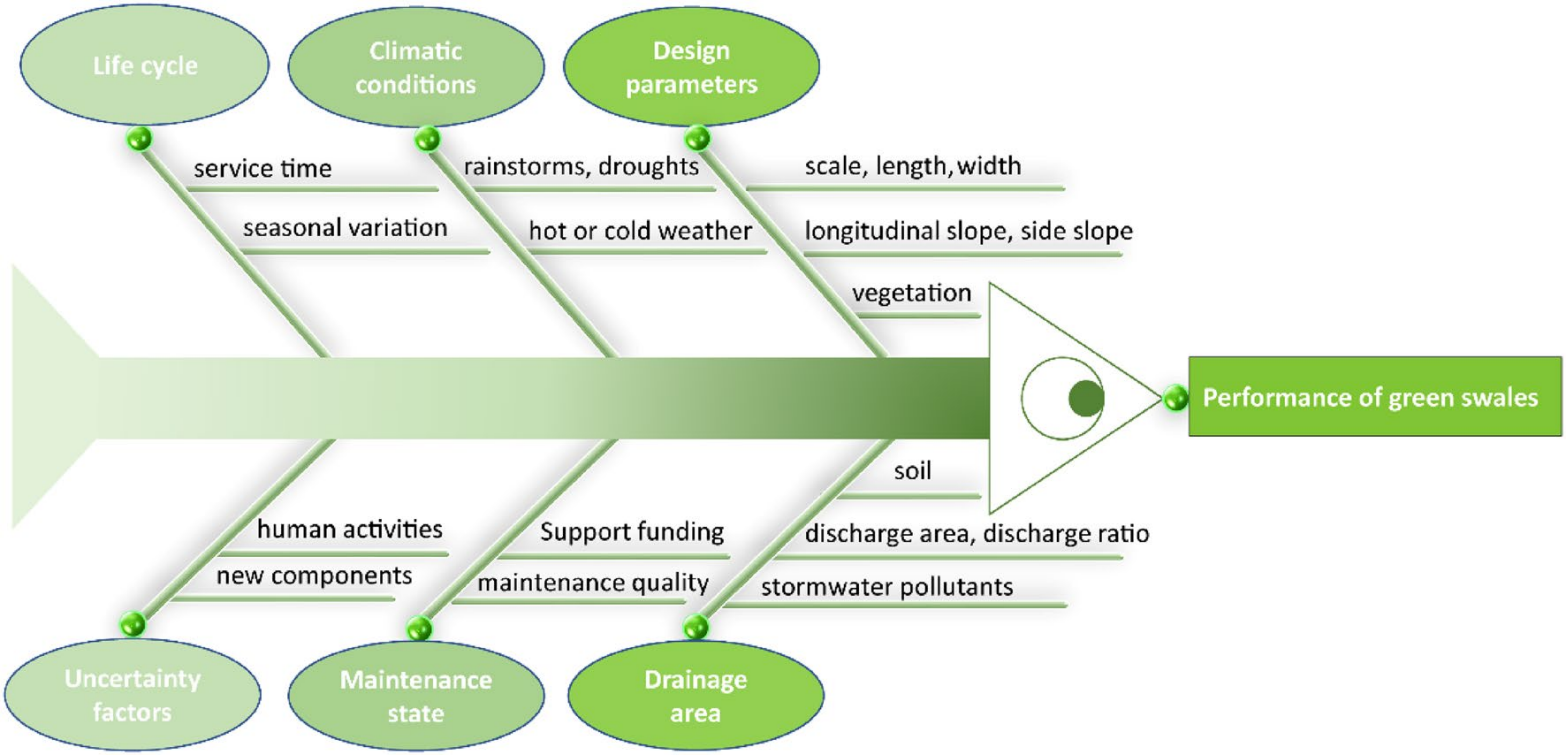
Potential influential factors of green swale performance (An Ishikawa diagram, also known as the fishbone diagram. The closer the influencing elements are to the fish head, the more impacts they have on the green swale).

Urban drainage systems can be affected by particulates and dissolved materials (e.g. roadside dusts). Nutrient concentrations can be high from inputs of organic materials and wastewater outflows. Swales are a potentially sustainable option for pollution reduction^19^. The performance of green swales in removing pollutants is affected by their design, drainage area, climatic conditions, maintenance status, lifecycle, and other factors (Fig. 1)^14,16,20–22^. The coverage of green swale construction is one of the most significant influential factors that control their performance. Local climate is also a considerable control on the elimination of elevated nutrients in green swales, partly because of its impact on the colonisation and growth of vegetation^23^.

The effectiveness of SCP facilities in the context of future climate change has also not been sufficiently studied. Current knowledge and practice of SCP facilities is limited in their ability to comprehensively address climate challenges, particularly in the aspect of climate change adaptation^24^. Previous studies have concentrated on assessing the attitudes of residents towards SCP facilities as a means of adapting to climate change^25^, reconceptualizing SCP for climate change adaptation^26^, and quantifying the environmental effects of both SCP and traditional facilities through lifecycle assessment^27^. Assessing the performance of SCP facilities under existing climatic conditions and anticipated future climatic scenarios could help identify limitations in how these facilities function, with important implications for effectively addressing climate challenges. It would also provide empirical support for planning and optimising the enlargement of SCP facilities to better align with the demands of adapting to climate change. There is a pressing need for the development of regional and national scenarios to assess the mitigation potential of SCP in the context of climate change^28^. Many SCP interventions will need to address local challenges and be tailored to site-specific temporal, spatial, and functional dimensions^29^. Indeed, SCP has been defined as “place-based partnerships between people and nature” for climate change adaptation^30^, implemented in part by local communities and providing community-led protection^28^. The distinction between SCP evaluations conducted at the city and community levels is that the former provides the overall performance of sponge cities and is typically generalised given the large area of focus, whereas focus at the community scale is more specific to the spatial area of focus, and attuned to community-level issues and design. To attain the genuine ability to adapt to climate change, SCP implementation needs to be coupled with urban greening initiatives and involved with local communities^31^.

Our overarching aim of this study, which targeted to explore how the effectiveness of SCP facilities may change with climate change, and how increasing the coverage of bioswales may offset urban runoff reductions in efficiency in the future. Consideration was given to both water quantity and water quality reductions in bioswales.

To achieve these aims, data from Cicheng New Town in Ningbo, China, was used to develop a model using the storm water management model (SWMM, issued by the United States Environmental Protection Agency)^22^. The performance of bioswales in combination with other SCP facilities, under future climate change scenarios, was assessed following three specific analyses:

1. An analysis of how different rainfall intensities (i.e., 2, 5, 10, 20, 50, and 100 years return period) and precipitation patterns (i.e., early peak, central peak and late peak) affect the performance of bioswales;
2. Quantification of the performance of existing SCP facilities in the study area under different climate change scenarios (i.e., rainfall intensity and consecutive dry days);
3. Identifying suitable coverage of bioswales within the study area for climate change adaptation.

## Results

### The impacts of rainfall intensity and distribution on urban bioswales

**Figure 2:**
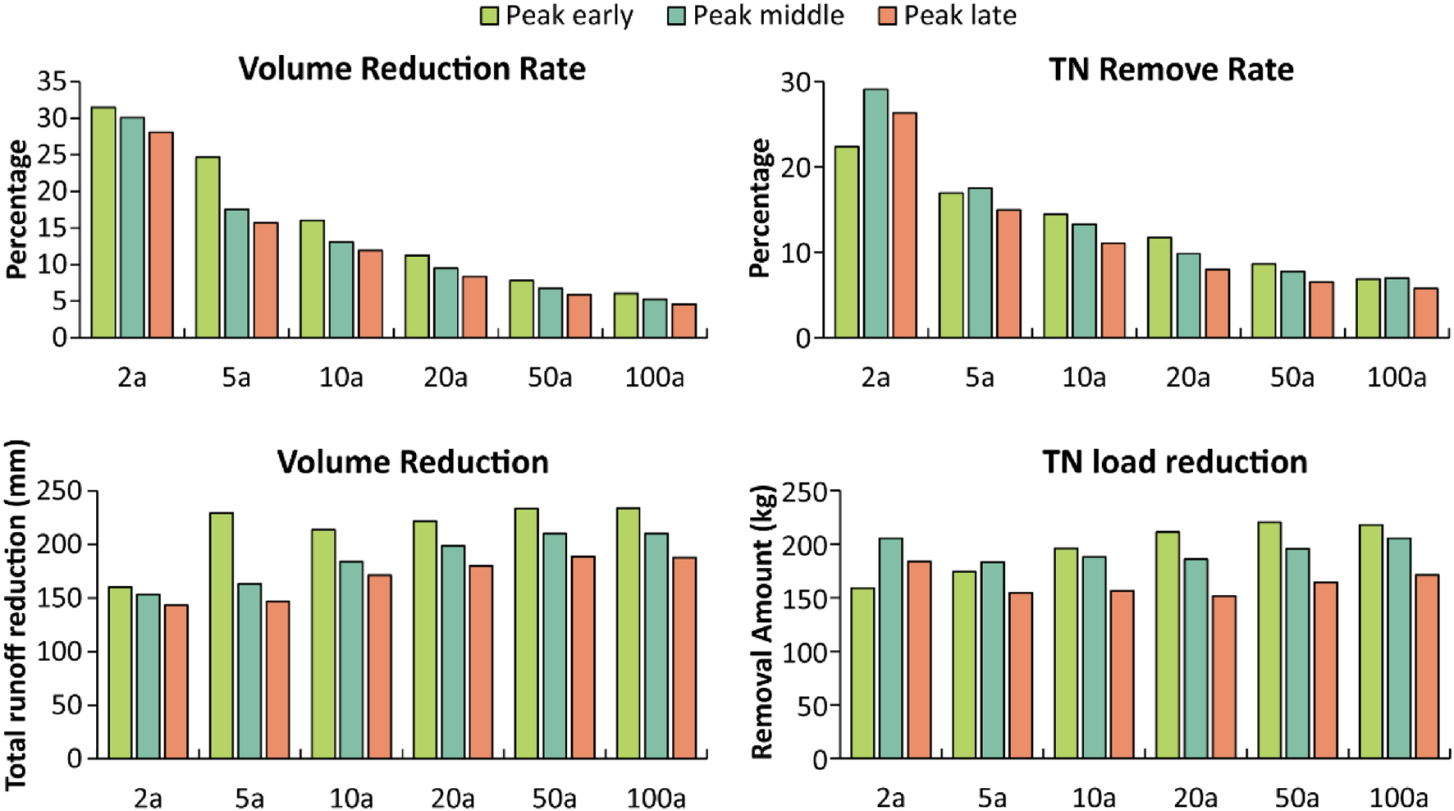
Performance of bioswale in terms of volume reduction and total nitrogen load reduction.

The total runoff volume reduction and the total amount of nitrogen removed from bioswales increased as rainfall intensity increased under all scenarios, but the relative reduction as a percentage of total rainfall typically decreased (Fig. 2). In other words, a smaller proportion of the total precipitation was conveyed via bioswales as rainfall events increased in magnitude, as would be expected. Whilst runoff volume and nutrient loads both decline, there is not a strong correlation between runoff volume and nutrient loads for different rainfall patterns and event intensities.

In general, bioswales performed best when the rainfall peak occurred earlier in the event, and were less effective when the rainfall peak occurred later in the event across all scenarios. Regardless of rainfall pattern, the trend of effectiveness versus event magnitude was asymptotic. Runoff reductions asymptote between 1-in-10 and 1-in-20-year events, with only small increases in the total runoff reduction occurring as event magnitude increased further. Across three different rainfall patterns, runoff reduction as a percentage of the total rainfall declined from approximately 30% for a 1-in-2-year event to 8% for a 1-in-20-year event, declining to 6% for a 1-in-100-year event. An exception is the reduction in total runoff for 1-in-5-year events when the rainfall peak is left-skewed (i.e., the peak occurred early in the event), which is greater than for 1-in-10 and 1-in-20-year events.

Total nutrient loads were also reduced across all scenarios. However, trends were not as clear as runoff volume and rainfall pattern impacts had a greater impact on how bioswale effectiveness changed with rainfall magnitude. For left-skewed events, total nutrient load reduced as event magnitude increased, with an asymptote around 1-in-20-year events. However, for rainfall with a central peak and late peaks, total nutrient load reductions were consistent for all event magnitudes. When presented as a proportion of the total rainfall, nutrient loads began to plateau around 1-in-5 to 1-in-10-year events.

### Potential of SCP facilities to address urban runoff risks under climate change scenarios

Rainfall data observed on 14 September 2022 was used for the simulation of the three distinct climate scenarios, including a middle-of-the-road (SSP2-4.5), dark future (SSP3-7.0), and strange one (SSP5-8.5). Simulation of each climate scenario included a comparative analysis of the absence of SCP facilities and the presence of SCP facilities in the study area. Under all three climate change scenarios, there was little difference in runoff reduction due to SCP facilities, which ranged between 8.2 and 8.6% of the total precipitation (Table 1). This was approximately 60% lower than for equivalent value for the baseline (contemporary) conditions.

**Table 1:** Modelling results of three climate scenarios in terms of urban runoff reduction and total nitrogen reduction.

Scenarios	SCP performance of different scenarios			
	Runoff reduction rate (%)	Ratio (%)	Total nitrogen removal rate (%)	Ratio (%)
Baseline (SCP)	14.33	100	24.15	100
SSP2-4.5 (SCP)	8.54	59.6	17.93	74.2
SSP3-7.0 (SCP)	8.63	60.2	18.12	75.0
SSP5-8.5 (SCP)	8.18	57.1	17.53	72.6

A similar pattern was noted for nitrogen removal rates, which dropped from 24% for baseline conditions to approximately 18% under climate change, with all climate change scenarios having a similar impact on nitrogen removal (Table 1). This is hypothesised to be because even under the middle-of-road climate scenario (SSP2-4.5), the capacity of SCP facilities is exceeded. So, although other scenarios represent more intense precipitation, they do not have an additional impact on the SCP effectiveness.

### The required coverage of bioswales to offset climate change

The bioswales implemented in our research area are part of China's Sponge City Program, and they have been designed to cope with 1-in-5-year events, following national legislation. Modelling results indicate that the current SCP provision successfully achieves this goal. However, the findings also suggest that the existing coverage of SCP facilities is ill-equipped to address the challenges posed by forthcoming climate change.

Simulations increased the coverage of bioswales from 0.3% of the catchment to 4% by building newly similar bioswales in other places (the specific scale and location of bioswales in each catchment are shown in Table S2). Relevant results including total runoff and total nitrogen load under these scenarios are included from Tables S3 to S6. As expected, as bioswales increased in coverage there was a linear decrease in runoff volume and nitrogen loading.

To maintain the same level of runoff reduction as is currently afforded by bioswales under contemporary conditions, bioswales need to be increased in coverage to cover 4% of the catchment area, in comparison to their current coverage of 0.3%. To have the same proportional decline in nitrogen loadings, bioswales would need to cover at least 2% of the catchment area (Table 2).

**Table 2:** Simulation results of three climate scenarios in terms of urban runoff reduction and total nitrogen reduction under different coverages of bioswales.

Scenarios	Bioswale performance of different scenarios	
	Runoff reduction rate (%)	TN reduction rate (%)
Baseline (SCP)	14.33	24.15
SSP2-4.5 (Bioswale-1%)	10.41	22.38
SSP3-7.0 (Bioswale-1%)	9.61	22.60
SSP5-8.5 (Bioswale-1%)	9.12	21.94
SSP2-4.5 (Bioswale-2%)	11.13	25.18
SSP3-7.0 (Bioswale-2%)	11.24	25.43
SSP5-8.5 (Bioswale-2%)	10.67	24.69
SSP2-4.5 (Bioswale-3%)	12.98	27.37
SSP3-7.0 (Bioswale-3%)	13.10	27.62
SSP5-8.5 (Bioswale-3%)	12.45	26.80
SSP2-4.5 (Bioswale-4%)	14.96	29.40
SSP3-7.0 (Bioswale-4%)	15.10	29.66
SSP5-8.5 (Bioswale-4%)	14.37	28.81

## Discussion

### Findings of this study

As rainfall intensity increases, total runoff volume reductions increased due to the presence of bioswales; however, the reduction rate decreased with rainfall intensity (Fig. 2). Bioswales were most effective when the rainfall peak occurred early in the event, and were less effective when peak rainfall occurred later within events (Fig. 2). Bioswales typically reduced runoff by 30% for 1-in-2-year events and reduced nitrogen loadings by 30%. As rainfall intensity increased beyond a 1-in-5-year event, especially with central and right-skewed peak rainfall, bioswales exhibited poorer performance, reducing runoff by between 10 and 6%.

Given this insight, we utilized observed rainfall data to simulate a 100-year return period rainfall event with a delayed peak with SCP facilities in the study area under different future climate scenarios. The performance of SCP facilities decreased to approximately 6% for both water reduction and nutrient removal under all three climate change scenarios (Table 2). Increasing the coverage of bioswales so that they cover 4% of the catchment area would offset the reductions in performance due to climate change.

### Interpretations of key findings

#### Performance of bioswales under different rainfall patterns

In the context of various rainfall intensities, the overall nitrogen removal rate and runoff reduction rate of bioswales exhibited a diminishing trend as the rainfall intensity increased. This aligns with other studies that suggest that when rainfall events have a low return period (indicating a high frequency of occurrence), SCP facilities tend to excel in performance, offering heightened effectiveness in mitigating the adverse effects of rainfall events^32,33^.

Bioswales demonstrated their highest effectiveness when encountering early rainfall peaks, while their performance significantly declined when faced with late peak rainfall events. This might be because an earlier peak could activate the swale’s drainage and retention functions earlier in the event, effectively slowing down the rate and peak flow of stormwater runoff.

In other words, where peak flows are late in an event, the earlier rainfall may saturate bioswales, reducing their ability to infiltrate the peak flow and reducing their effectiveness at reducing nutrient loads. In contrast, an earlier peak flow is partially infiltrated and denitrified by the bioswale at the start of the event. Recent modelling studies have shown that the occurrence of later rainfall peaks creates the largest peak flows at catchment outlets, while mid- and early-peaked rainfall patterns exhibit progressively lower peak flows^34,35^, supporting our findings.

The "*Ningbo Sponge City Planning and Design Guidelines*" released by the municipal government of Ningbo in 2019, stipulated that the design recurrence interval of rainwater pipelines and channels in important regions should reach the level of 1-in-5 years. Bioswales were suitably sized and designed for efficient water volume reduction in the study area. SCP facilities have generally been found to achieve optimal performance when designed to manage precipitation events that align with a 5-year return period^18^.

Across the Pacific, similar NbS in the Berkeley neighbourhood in northwest Denver, Colorado (meets the flood control requirements for a 5-year return period storm event, but falls short of complying with the flood control standards for a storm event with a 100-year return period^36^. Total nitrogen reduction in bioswales takes time to complete and, therefore, nitrogen removal requirements would be expected to decrease as rainfall intensity increases because flow velocity increases^37^. Although not a factor in our modelling approach, heavier downpours could also wash away sediment and infiltration layers in swales, decreasing the effectiveness of nitrogen removal.

#### Performance of SCP facilities under climate change scenarios

The performance of SCP facilities (i.e., bioswales, permeable pavements, and rain gardens) in the study area under three different climate change scenarios showed nearly identical performance. The performance of SCP facilities in terms of water quantity and quality declined from 14 and 24% to roughly 8% and 18%, respectively, under the impacts of three climate change scenarios.

The reason for this is that the rainfall that was recorded on September 14, 2022, was an occurrence that only happens once every 100 years, whereas the study area's rainwater pipes and channels were designed for events that occur once every 5 years. As a result, rainwater overflow forced the SCP facilities in the study area to operate at maximum capacity.

#### Suitable bioswale coverage for climate change adaptation in the future

Expanding the spatial extent of bioswales within the study area could be an effective response to climate change. The capacity of bioretention surface storage plays a pivotal role in determining the system's resilience to elevated rainfall^38^.

In London, investigations have found that the extent, types and distributions of greenery needed to realize significant urban economies of scale are intricate, primarily due to a lack of clarity in the necessary degree of expansion^39^. Based on the simulations conducted in this study, two adaptation options have been proposed, focusing solely on the incorporation of bioswales to mitigate the effects of future climate change for maintaining the current levels of urban runoff reduction.

The first option involves expanding the bioswale area to 2% of the catchment area, effectively addressing climate change's influence on runoff quality. This is in line with the findings of Melbourne research, which found that the best-designed biofilter covered at least 2% of the catchment area^40^. The second option suggests a minimum expansion of the bioswale to 4% of the catchment area, aiming to simultaneously mitigate climate change's impacts on water quantity, encompassing factors such as total nitrogen and runoff volume within the study area. It is important to note that these findings are specific to the study area; nevertheless, they offer valuable insights for similar research conducted at other community scales.

### Implications of the study

Whilst the study is focused on a single community area, it has wider relevance to areas beyond this case study. Climate change is addressed in 42% of the water-related studies focused on the impacts of SCP; however, it is acknowledged that there is still a lack of specific methods for providing a quantitative scientific evidence base^41^.

To provide crucial technical support for the reasonable planning of SCP performance under climate change, this study used SWMM to quantify the effectiveness of SCP in addressing urban runoff under current and future climate change. A similar approach could be undertaken elsewhere to provide similar quantitative data on the current and future effectiveness of swales of differing designs.

Furthermore, our findings demonstrated that the effectiveness of local SCP was significantly impacted by different rainfall patterns. To assess how well local SCP can handle urban runoff issues, it is vital to consider localised rainfall intensities and distributions. These findings can be used to support urban decision-makers in their planning and design for climate change adaptation from both planning and policy perspectives. For example, bioswales are more effective in areas where the community commonly suffers left-skewed rainfall events. Furthermore, given limited funding and consideration of the local circumstances (i.e., the severity of nitrogen pollution or runoff volume problem), urban planners can determine the minimal scale of bioswale construction required to meet the needs of climate change adaptation. China does not yet include climate change adaptation in the requirements for building sponge cities from a policy standpoint. To satisfy the need for climate change adaptation, we propose that future decision-makers include in their policy framework the minimal climate change adaptation coverage for developing different sponge city infrastructure or the pertinent water quantity and quality control indicators.

Integration of bioswales with current urban infrastructure is a significant challenge. To tackle this difficulty, three approaches including "flexible design"^42^, "interconnected SCP network"^43^ and "community engagement"^44^ are recommended. In this study, "flexible design" could represent the conversion of bioretention cells from bioswales into modular units that are designed to suit the various site constraints and space conditions seen in high-density communities. The approach taken allows investigation of “interconnected SCP network” denotes, considering how bioswales remain connected, creating an interconnected green network space. To guarantee the long-term performance stability of bioswales, “community engagement” is required. Presenting options to communities using a modelling approach as demonstrated here could help such engagement, and the education of residents in the importance of green systems in their communities. By implementing these strategies, bioswales can be integrated into highly developed or densely populated communities, improving resilience to the effects of climate change, and promoting sustainable stormwater management.

Based on a recent cost–benefit analysis study on SCP facilities^45^, bioswales have a total unit cost of 1580.39 RMB/m^2^. We estimated that an increase of 2% in bioswales would take about 1.6 × 10^8^ RMB, and an increase of 4% in bioswales would cost about 3.2 × 10^8^ RMB in Cicheng New Town. The necessary expenditure is high, but the advantages to society, the economy, and the environment are also substantial^45,46^. Past studies in Ningbo have explored community perceptions of and engagement with NbS, finding that future improvements in perception and engagement were likely to be substantial and that raising public awareness of SCP practices could encourage stronger co-production^47^. Increased community acceptance of bioswales can be achieved via effective communication and educational initiatives that help clear up misconceptions and foster trust. Active community involvement is essential for sustaining public support and guaranteeing the ongoing SCP facilities.

### Other factors of bioswales for climate change adaptation

Long-term droughts before rainstorm events can have a significant impact on the concentration of pollutants that accumulate in urban runoff, which directly affects the efficiency of SCP. For instance, the infiltration system of swales may get clogged by the deposition of surface sediments washed into the system by intense rainfall, reducing the performance of bioswale functions^48^. Good maintenance management is therefore essential for SCP to sustain adequate performance over time. To enable the good maintenance management of bioswales (e.g., routine inspections, vegetation control, and soil amendment) in response to climate change and urban development pressures, ongoing capital investment is required. However, funding often faces challenges and should not rely solely on one source of capital investment such as direct government investment but should carefully consider all possible financing options^48^.

Local vegetation can more readily adapt to the soil and climatic conditions in the local area, which is important given the challenging environment that swales provide (e.g., periods of water scarcity followed by complete submergence). Establishing healthy vegetation in swales is important but requires careful consideration of plant species and the functions that the vegetation needs to provide. In China, cold and drought-tolerant vegetation such as *Ligustrum lucidum* and *Ophiopogon japonicus* are planted in bioswales^48^. Compared to other plant species including *boxwood*, *ryegrass*, *shiny privet*, and *Chlorophytum comosum* “Variegatum” or having no plant at all, these plants have been shown to increase water reduction in bioswales more effectively^20^. However, relevant work on the role of vegetation in swales is limited and there is little information on the effectiveness of vegetated swales in managing water quantity and water quality under various climatic conditions, and in association with climate change. We recommend future studies should focus on how different vegetation species affect the infiltration and pollutant removal capabilities of bioswales.

Based on an analysis of 59 swales, the geometric design elements of different swales, such as length, centreline slope, slope, and emission ratio, were found to be unrelated to the removal rate of most pollutants^21^. There is a propensity to achieve higher removal rates for dissolved nutrients and some particulate pollutants when the geometric design of swales (e.g., increasing width or depth) increases hydraulic retention time^21^. However, deepening the bioswales may have an impact on the groundwater, and widening bioswales may affect traffic safety. To assess the viability of these solutions, each of them needs to take additional considerations into account. Therefore, we believe that it is more feasible to adapt to climate change by increasing the coverage of bioswales with the existing same specifications.

## Conclusion

This study explores the effectiveness of bioswales under contemporary and future conditions, taking an urban community in Ningbo, a Chinese pilot Sponge City as an example. The conclusions of this study are: (1) bioswales were most effective in scenarios when the rainfall peak is left-skewed, while their performance is least effective in scenarios when the rainfall peak is right-skewed. (2) The overall performance of existing SCP facilities (bioswales, rain gardens, and permeable pavements) in terms of runoff volume reduction and nutrient removal is equivalent when considering the three mild to pessimistic climate change scenarios when the rainfall peak is right-skewed. The effects of climate change are thought to be more severe than these SCP facilities can mitigate. (3) To address the impact of climate change over 20 years from 2020 to 2039, increasing the community's bioswale coverage to 2% is a viable solution for the removal of nutrients from urban runoff, whereas expanding the community's bioswales to at least 4% would effectively address both flooding and water quality issues simultaneously.

Overall, relevant methods in this study are applicable for designing new NbS systems in other similar community settings, while offering a NbS for climate change adaptation. We recommend that other similar urban communities take rainfall patterns into account when planning the coverage of NbS. In addition to the intensities of rainfall, consecutive dry days are a significant contributing factor to pollution from urban runoff in the future climate. This study presents evidence that sheds light on how well NbS performs in reducing urban runoff in the context of climate change. Future studies would be more meaningful if the coverage of NbS could be designed with cost-effectiveness in mind and comparative analyses among different NbS options would provide a more comprehensive understanding of their efficacy.

## Materials and methods

### Modelling approach

SWMM is a dynamic hydrological modelling tool designed for the simulation of runoff and water quality in urban environments, applicable to both single-event and continuous simulations. From a functional standpoint, it comprises three principal modules: simulation for quantifying water flow, simulation for assessing water quality, and the incorporation of SCP facility modelling. We first filled the knowledge gap about how rainfall patterns affect bioswales by using the SWMM. We ascertained the performance variance of the current SCP facilities between the climate change scenarios and the baseline scenario by simulating these scenarios. Under the identified rainfall pattern that bioswales perform worst, the lowest increased coverage with bioswales for satisfying the demands of protection level of current SCP facilities was determined.

In the first stage, we created a model of the Cicheng community in Ningbo, China, to analyse how different rainfall intensities (i.e., 2, 5, 10, 20, 50, and 100 years return periods) and precipitation patterns (i.e., peak early, peak middle and peak late) affect the functionality of bioswales. The urban runoff reduction potential of NbS for climate change adaptation was identified in the second stage. Existing SCP facilities including permeable pavements, rain gardens and bioswales were modelled in the study area. The scale of each facility in each catchment area is documented in Table S7. A study of similar facilities (i.e., rain barrels, grass swales, bioretention cells, and permeable pavements) under climate change conditions in a residential neighbourhood in London, Ontario, Canada, showed that all facilities demonstrated varying degrees of degradation in their performance under climate change, with bioretention cells being the most resilient^49^. Thus, this study primarily focused on bioswales, as a type of bioretention cell. The bioswales in the study area are clustered in the downstream area of the site, offering the opportunity to explore the potential for increased bioswale coverage. As such, the coverage of bioswales was altered between treatments, but other SCP facilities remained unchanged.

**Figure 3:**
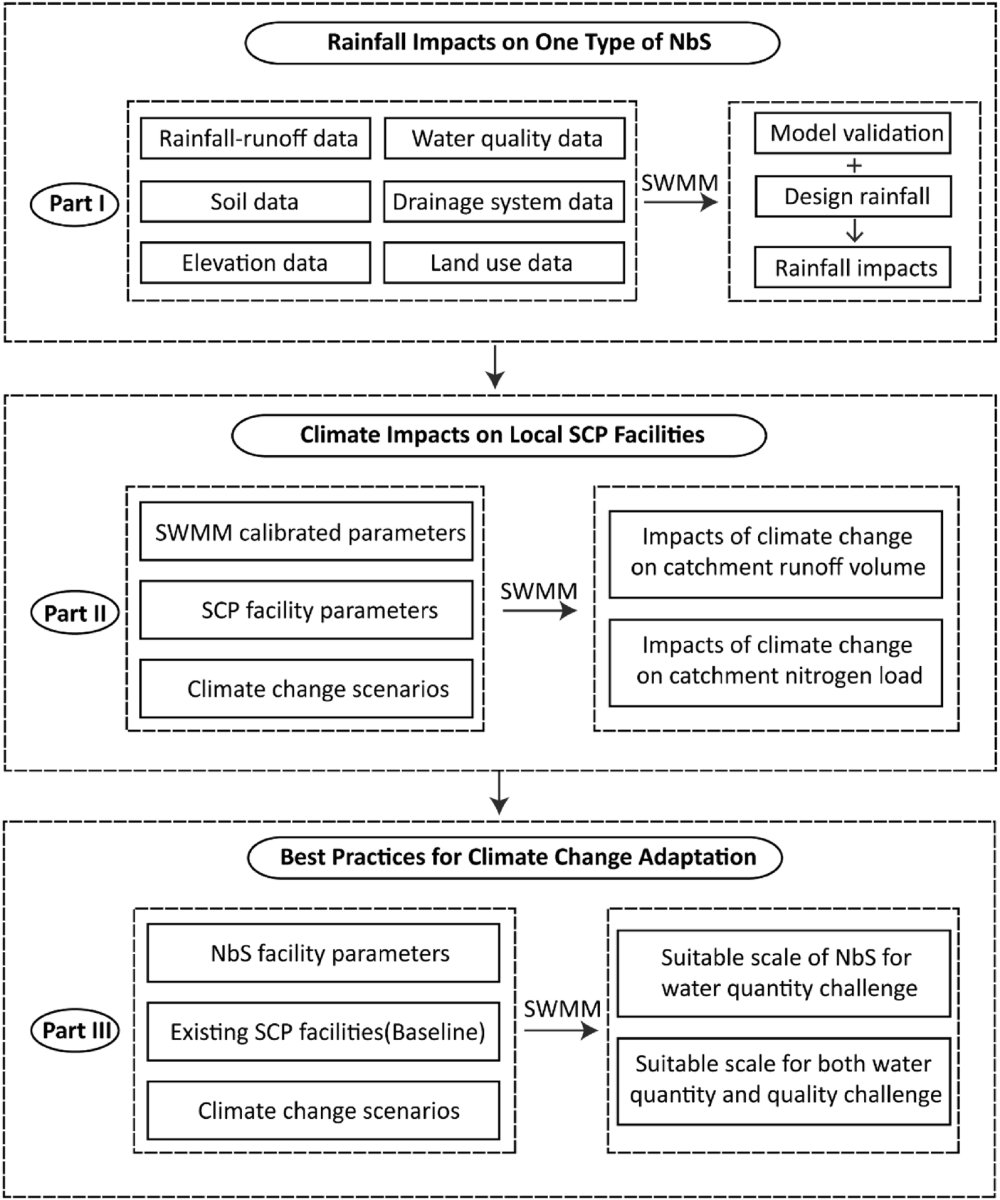
A flow chart for NbS coverage design towards climate change adaptation.

To change the coverage of bioswales in the study area, more bioswales were added into locations where they were not previously present to increase their land area coverage within the area. This allows the geometry of bioswales to remain unchanged. The increased coverage of bioswales is shown in Table S2 of the Supplementary material. The newly added bioswales located in each sub-catchment were increased in an equal proportion as designed. When the area of bioswales in the existing sub-catchment area was less than the designed target value, bioswales were added to the catchment. By evaluating the model results, we explored how rainfall pattern affects NbS performance, with implications for the required coverage of NbS needed to mitigate future climate impacts (Fig. 3).

### Study area

**Figure 4:**
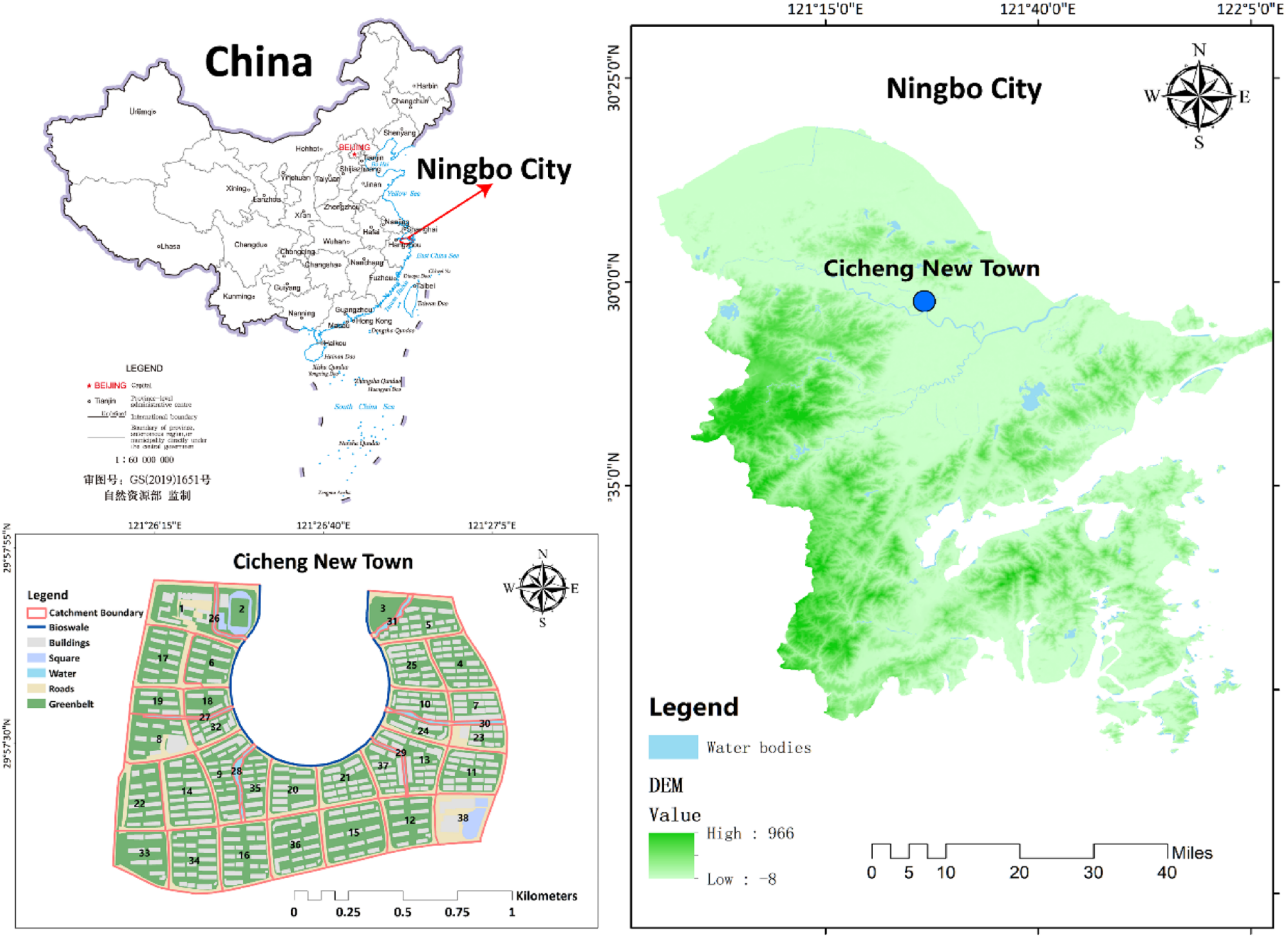
Location of the research area and relevant information. In this figure, the map of China was obtained from http://bzdt.ch.mnr.gov.cn/, the website of the Chinese government for public use. The elevation data (DEM) was obtained from Ningbo Planning and Geographic Information Centre in China.

Cicheng New Town (Fig. 4), located in Ningbo, China, was selected as the study area. Ningbo, a Chinese Sponge Pilot City, is located on the southeast coast of China with longitude and latitude ranging from 120° 55′ to 122° 16′ E, 28° 51′ to 30° 33′ N. The elevation of Ningbo is generally high in the southwest and low in the northeast. Ningbo has a subtropical monsoon climate, with an overall increasing trend in annual precipitation and precipitation intensity, and a declining trend in annual precipitation days. According to the climate summary released by the Ningbo Municipal Government (https://nb.zj.weather.com.cn/weather/weatherStatic/climateNingbo/index.html), the yearly average temperature is 16.4 °C, while the annual average precipitation is 1480 mm. Specifically, rains occur in Spring from March to June and typhoon rains from August to September comprise the major rainy season. Of the total yearly rainfall, 60% comes from precipitation between May and September.

The community of Cicheng New Town occupies a space of around 5.2 km^2^, and waterlogging is primarily caused by low-lying terrain. This community is a high-density urban residential area in China, with a planned population of 85,000 with infrastructure including schools, hospitals, and wetland parks. The land has low topography in the centre and high terrain on the east and west borders. Before the community was built, there were numerous rivers and streams in the area, and the predominant land uses were rice fields, villages, vegetable cultivation, and aquaculture. After constructions, runoff pollution caused by rainstorms also poses a threat to nearby water bodies, such as the central lake in the Cicheng New Town. To effectively combat the risks of urban runoff, the community built bioswales for stormwater from plots and roads to flow into an ecological filter belt installed along a river or road, where it enters a permeable pipe for stormwater collection after infiltration.

Besides, SCP facilities including rain gardens and permeable pavements were also built in the community to store and purify urban runoff. After that, the stormwater flows into the main rainwater pipe, which relates to the river channel inside the community, and finally flows into the central lake. The generalization of the research area mainly includes sub-catchment areas, drainage networks, pipe junctions and the outfall.

### Data collection

Data for inclusion in the SWMM model included the land use type, drainage system, rainfall event, water quality, soil type, and elevation data (see Table S8). Table S9 displays the percentage of each type of land use throughout the whole research region. Each land use type was given a standard runoff coefficient and event mean concentration (EMC) value of total nitrogen (Table S10). The drainage data was used for the hydrological simulation and rainfall data and water quality data (i.e., total nitrogen values of EMC) were input data in simulations.

Among five popular probability distribution analysis techniques, the best-fitting probability distribution is the Pearson III type distribution analysis^50^. Thus, Pearson type III distribution was used in this study for calculating ${R}_{Design}$ (formula 1) during 24 h based on the observed data from Yinzhou Rainfall station near this study area from 1983 to 2019 (Table S11). The relevant P III frequency curve fitting diagram is shown in Fig. S2.

Results including design rainfall and magnification ratio ($K$) are shown in Table S12 of the Supporting Material. The rainfall observed on 14 September 2022 as well as pertinent data with varying rainfall intensities (i.e., return periods of 2, 5, 10, 20, 50, and 100 years) produced using the same frequency amplification method^51,52^ (formula 1) based on the observed data. Observed rainfall and related rainfall data during 24 h in different return periods are available in Table S13. Notably, typhoon “Muifa” on September 12, 2022, created an extraordinary episode of high rainfall, which is what we measured for our rainfall data. On that day, Ningbo experienced a once-in-a-hundred rainfall event with an average of 291 mm of precipitation across the entire city.

1 $$K= \frac{{R}_{Design}}{{R}_{Observation}}$$

In formula 1, ${R}_{Design}$ is the designed total rainfall value under different return-period rainfall events. ${R}_{Observation}$ demonstrates the observed total rainfall value.$K$ represents the ratio of design rainfall ${R}_{Design}$ to actual rainfall ${R}_{Observation}$. The observed rainfall data during 24 h with Muifa Typhoon on 14 September 2022 is 292.6 mm.

Based on an existing rainfall event (a right-skewed distribution, Table S13), this study created another two sets of rainfall data with the same total rainfall but different patterns to identify the impact of rainfall distribution on the bioswale performance: a left-skewed distribution and a central peak distribution (Tables S14 and S15). Soil data was used to calculate the infiltration properties, including soil capillary suction head, soil saturated hydraulic conductivity and initial deficit. Elevation data was used to calculate the slope properties of the catchment area. The coverage of SCP facilities presented in the study (including bioswales, rain gardens, and permeable pavements), as well as other attributes of these facilities, were also included (see Tables S7 and S16).

### SWMM construction

#### Area generalisation

The methods for sub-catchment generalization can be divided into two categories: hydrological methods based on digital elevation models (DEM) and geometric methods based on nodes. The former is more frequently employed, and its basic principle is to divide the watershed and determine its flow direction using DEM and the D8 (deterministic-8 node) algorithm^53^. This approach considers how natural terrain affects the direction of water flow, ignoring the influence of roads and buildings on rainwater runoff in urban catchment areas.

The latest hydrological method utilizes auxiliary data such as roads, buildings, and drainage systems to correct sub-catchment areas based on DEM partitioning^54^. Hydrological methods based on DEMs integrated with land use data and drainage data were used in this study with the following guidelines: (1) The catchment region's river channel should be a separate sub-catchment area; (2) Clustered buildings separated by roads or rivers should be in the same sub-catchment area; (3) Individual buildings should be located within the same sub-catchment area. After adjustments were made to meet these three guidelines, it was necessary to perform topology checks in ArcGIS software on sub-catchments and make further adjustments where necessary. The specific topology rules are as follows: (1) An area must not overlap another area from the same layer; (2) A void cannot exist between areas in the same layer. We modified the errors found in the topology check inspector until the topology requirements were met.

#### Scenario design

**Figure 5:**
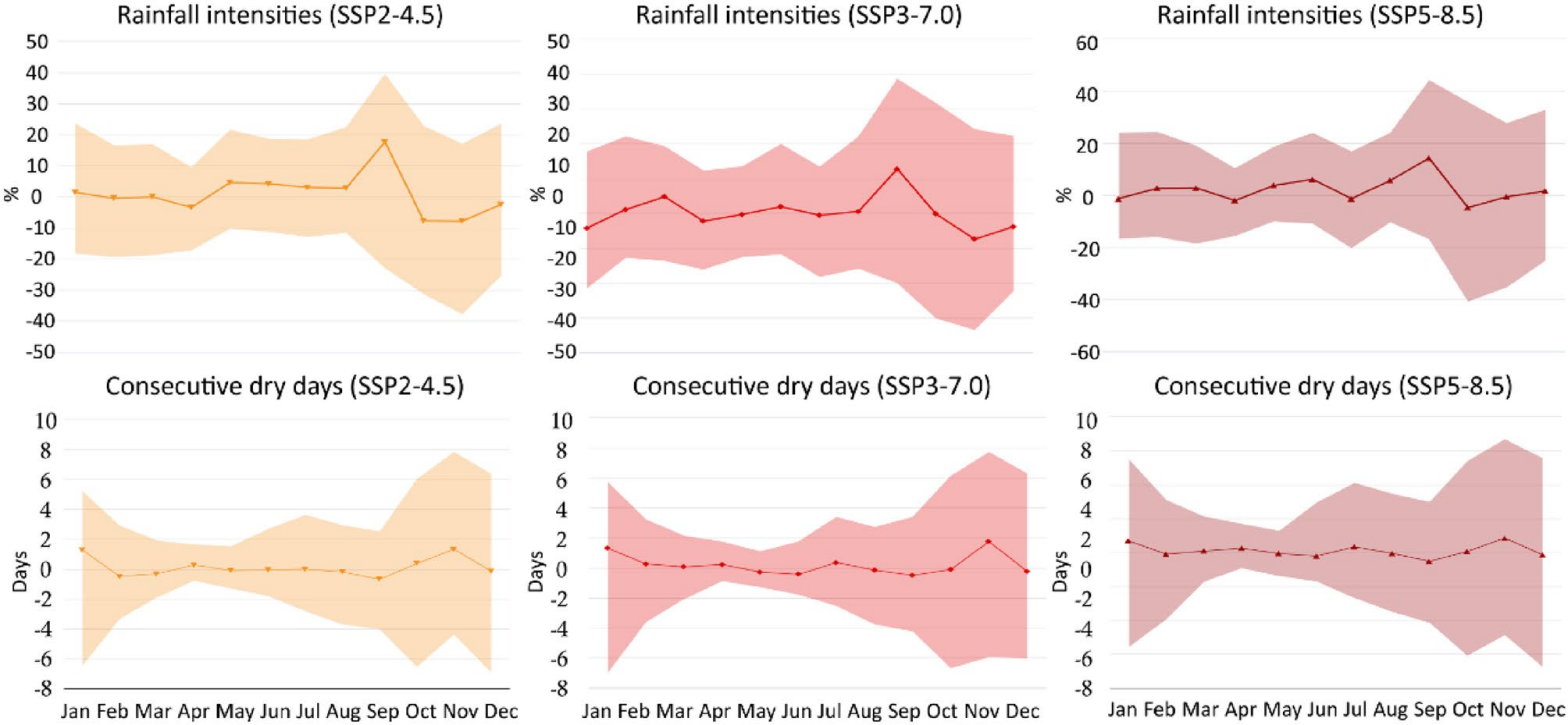
Precipitation Percent Changes of rainfall intensities and maximum number changes of consecutive dry days for SSP2-4.5, SSP3-7.0, and SSP5-8.5 scenarios in each month for 2020–2039 (Zhejiang Province, China), sourced from climate change knowledge portal of the World Bank Group for Zhejiang Province, China (https://climateknowledgeportal.worldbank.org/country/china/climate-data-projections). Based on the relevant data from the period: 1995–2014 with multi-model ensemble.

Three main components make up the design of simulation scenarios: the true representation of SCP facilities under a real rainfall event, the true representation of SCP facilities under climate change conditions (where the coverage of bioswale is 0.3% of the research area), and increasing the area of bioswale in catchments under climate change conditions to determine their suitable coverage that maintain current levels of urban runoff reduction. For comparison with other scenario simulations, the initial section of the simulation was used as a baseline scenario. Scenario Model Intercomparison Project (ScenarioMIP) adopted by the latest coupling model, CMIP6 based on the Shared Socio-Economic Pathways (SSPs), can be used to accurately evaluate the impact of climate change on different land uses and provide reliable simulation results for predicting regional climate change^55,56^. The SSPs can be divided into five scenarios, two relatively optimistic scenarios (SSP1-1.9 and SSP1-2.6), the middle scenario (SSP2-4.5), the dark future (SSP3-7.0), and the strange scenario (SSP5-8.5). Recently, SSP2-4.5 and SSP5-8.5 scenarios were used to represent climate change spanning from moderate to high emission levels in a study on green roof adaptation to climate change^57^. Three scenarios including “a middle-of-the-road future (SSP2-4.5)”, “a dark future (SSP3-3.7)”, and “a strange one (SSP5-8.5)” were used to simulate the effects of climate change in this study (Fig. 5).

The Coupled Model Inter-comparison Projects, overseen by the World Climate Research Programme, provided the online model data from the global climate model compilations for climate prediction data in this study. It is based on training data from 1995 to 2014 and simulates climatic data using a multi-model ensemble.

This multi-model ensemble method represents the range and distribution of the most reasonable prediction results for climate system changes in the selected SSP. This multi-model ensemble method captures the range and distribution of the most plausible forecast findings for changes to the selected SSP's climate system. The primary components of the climate prediction data used in this study are the percentage changes in rainfall intensity and the maximum number of days with continuous drought. Tables S17 and S18 in the supporting material include the specific details.

According to the precipitation percent changes of rainfall intensities for SSP2-4.5, SSP3-7.0, and SSP5-8.5 scenarios in each month for 2020–2039 in the study area (see Table S17 in the supplementary material), the data in September exhibits the most pronounced influence of climate change on climate simulation data in the study when compared to data from other months. Consequently, based on the baseline rainfall data observed on 14 September 2022 and consecutive dry days with 7 days, we proceeded to simulate the three distinct climate change scenarios.

To assess the importance of the proportion of the catchment covered by bioswales to their performance, the coverage of bioswales was increased from 0.3% of the catchment area to 4%. This was achieved by adjusting the value of the area of bioswales in each catchment in the SWMM. The width of each bioswale was maintained at 2.5 m based on the actual conditions of existing bioswales to guarantee uniform performance of each bioswale per unit area. The specific areas of bioswales in each catchment are shown in Table S2 of the Supplementary Material. The location and scale of bioswales with 0.3%, 2% and 4% were shown in Figs. S3, S4 and S5, respectively.

#### Model evaluation

Two primary components of the model validation are the verification of the water simulation and the verification of the overall nitrogen removal rate in the research region. The comprehensive runoff coefficient for the research region serves as the validation target parameter for water quantity modelling. Relevant data of rainfall–runoff coefficients for different catchment area types are shown in the supporting material (Table S10).

The value of the comprehensive runoff coefficient was produced by weighting the rainfall-runoff coefficients for various types of catchment areas, based on empirical values of runoff coefficients for various underlying surfaces (green space, water system, commercial street, non-commercial street and rooftop). Formula 2 below displays the specific calculating process. Additionally, the validation goal values for the rate of total nitrogen removal in bioswales were based on measured values from studies in the same area.

2 $${\varphi }_{z}= \frac{\sum {S}_{i }{\varphi }_{i}}{S}$$

In Formula 2, the parameter ${\varphi }_{z}$ denotes the comprehensive runoff coefficient, while $S$ represents the cumulative area of the catchment. Additionally,${S}_{i}$ represents t ${\text{he}}$ specific area corresponding to individual catchment zones, and ${\varphi }_{i}$ characterized the comprehensive rainfall-runoff coefficient corresponding to different land use categories.

In this study, the root-mean-square deviation (RMSE) approach was used to verify simulation findings. RMSE is a statistical indicator of error goodness of fit index, which is used to measure the difference between model prediction value and observation value^58^. This method was used in this study along with the normalized objective function (NOF; Chow, Yusop and Toriman^59^) to verify the modelling results of the runoff coefficient of the research area and pollutant removal rate. Formulas 3 and 4 illustrate its calculation's underlying idea and method:

3 $$RMSE= \sqrt{\frac{1}{N}{\sum }_{i=1}^{N}{({X}_{ref}-{X}_{sim,i})}^{2}}$$

4 $$NOF= \frac{RMSE}{{X}_{ref}}$$

where ${X}_{ref}$ is the calculated reference value, ${X}_{sim,i}$ represents the simulated value. The optimum NOF value is 0, however, when site-specific data are available for calibration, values between 0.0 and 1.0 are acceptable^60^.

To verify the water quantity, the runoff was compared to the comprehensive runoff coefficient (i.e., the ratio of total runoff to total precipitation within the catchment area), which was calculated to be 0.51 (see Table S19). Modelled runoff under different rainfall intensities (i.e., 2, 5, 10, 20, 50, and 100-year return periods) approximated the comprehensive runoff coefficient, with RMSE and NOF values < 0.033 and < 0.644, respectively (Table 3).

**Table 3:** Model validation results in terms of runoff coefficient and total nitrogen removal rate.

Return period and total rainfall	Simulated value	True value	RMSE	NOF
2 years (67.30 mm)	0.5006	0.5100	0.0329	0.0644
5 years (105.34 mm)	0.5040	0.5100	0.0270	0.0530
10 years (131.67 mm)	0.5052	0.5100	0.0235	0.0462
20 years (155.08 mm)	0.5059	0.5100	0.0211	0.0415
50 years (184.34 mm)	0.5066	0.5100	0.0193	0.0379
100 years (207.75 mm)	0.5069	0.5100	0.0180	0.0352
Typhoon Muifa Sept 2022 (292.6 mm)	24.147%	24.15%	0.0030	0.0001

To validate the water quality, modelled results were compared to measured removal efficiency. We used actual rainfall data to verify water quality since rainfall has a substantial impact on the removal rate of total nitrogen. The total nitrogen removal rate was taken as 24.15%, which is an average of three field measures (26.02%, 23.66% and 22.76%) taken at the site between March and May 2019 (Table S20). The results of water quality validation are displayed in Table 3, and the validation procedure is depicted in Table S21. The NOF values are all near 0 and between 0 and 1, demonstrating the high flexibility relevant to validated model parameters.

The calculated comprehensive runoff coefficient was used as a validation value for repeatedly adjusting the uncertainty parameters. The rainfall data was selected from the Cicheng Rainfall Station on September 14, 2022. On that day, there was continuous rainfall for 24 h, with an hourly gap between each rainfall measurement. The calculated reference values and adjustment process are detailed in Table S22.

### Data analytics

The evaluation of SCP performance in mitigating runoff within the study area primarily relies on the simulation and comparative analysis of two scenarios: one representing the absence of SCP facilities and the other depicting the constructed SCP facilities in the study area. This assessment aims to quantify the extent of surface runoff reduction within each sub-catchment under conditions considering SCP facilities. The evaluation of the total nitrogen removal performance of SCP facilities in the study area hinges upon the simulation and comparative analysis of the mentioned two scenarios, specifically examining variations in total nitrogen concentrations within each pipeline. In other words, the quantified reduction in total nitrogen levels within pipelines of the research area serves as a direct indicator of the nitrogen removal capacity exhibited by constructed SCP facilities.

### Limitations of the study

Given the notable uncertainties inherent in global climate model simulations, this study utilized climate change simulation data covering the entire Zhejiang Province in China to project different scenarios at the community level in China. It is crucial to recognize that this method comes with certain limitations, particularly in accurately capturing spatial-scale rainfall patterns. As the effects of climate change vary across different regions, it is advisable to conduct similar studies in various locations to assess the localized implications on stormwater management^38^.

Moreover, the structural complexity of bioswale is compounded by the myriad of influencing factors that shape their performance. The SWMM bioretention cell module was used for bioswale modelling in this study, but this module ignores the effects of the slope of bioswales. Additionally, a sole consideration of rainfall and the duration of antecedent dry periods as climate change factors may not encompass the full spectrum of climate change impacts on the local environment, such as factors related to evaporation. The present study did not encompass a cost–benefit analysis related to the scalability of SCP facilities. Future research should consider this aspect, as it would offer a more comprehensive scientific basis for decision-makers when formulating plans for SCP facility expansion in the community.

There is a lack of analyses on other stormwater pollutants in urban runoff in this study despite evidence contaminants including microplastics, heavy metals, and bacteria can be eliminated by using bioswales^22,61^. Most of these runoff pollutants in bioswales can be simulated using a similar methodology described in this study. However, increased data support and tool development are required for future modelling studies with other runoff contaminants, especially microplastics where particle size, density, adhesion, adsorption, deposition, and resuspension all need to be considered within the modelling framework, not possible here.

### Supplementary Information

Supplementary Information.

## Data Availability

Relevant data during the current study are available from the corresponding author on reasonable request.
