# Supplementary material for: Harnessing the runoff reduction potential of urban bioswales as an adaptation response to climate change
Source: Sci Rep. 2024 May 28;14:12207. doi: 10.1038/s41598-024-61878-7 (PMC11133320; doi:10.1038/s41598-024-61878-7)
Supplement: Supplementary file 1 — Supplementary Information. [file 41598_2024_61878_MOESM1_ESM.pdf]

Supplementary Material

Harnessing the runoff reduction potential of urban bioswales as an adaptation response to climate change

Lingwen Lu[1,2], Matthew Johnson[3**], Fangfang Zhu[4**], Yaoyang Xu[2,5], Tian Ruan[2], Faith Ka Shun Chan[1,6**]

1. School of Geographical Sciences, Faculty of Science and Engineering, University of Nottingham Ningbo China, Ningbo 315100, China
2. Key Laboratory of Urban Environment and Health, Ningbo Observation and Research Station, Institute of Urban Environment, Chinese Academy of Sciences (CAS), Xiamen 361021, China
3. School of Geography, University of Nottingham, Nottinghamshire, NG7 2RD, UK
4. Department of Civil Engineering, Faculty of Science and Engineering, University of Nottingham Ningbo China, Ningbo 315100, China
5. Zhejiang Key Laboratory of Urban Environmental Processes and Pollution Control, CAS Haixi Industrial Technology Innovation Centre in Beilun, Ningbo 315830, China
6. Water@Leeds Research Institute, University of Leeds, Leeds LS2 9JT, UK

**Joint correspondence:

Matthew Johnson (m.johnson@nottingham.ac.uk)

Fangfang Zhu (fangfang.zhu@nottingham.edu.cn)

FKS Chan (faith.chan@nottingham.edu.cn)

Table S1 A standard terminology of each type of green swales with relevant functions[1,2]

Swale type	Definitions	Distinguishing features	characteristics
Grass swale	A grass swale is defined as an earth channel typically lined with turfgrasses designed to capture, convey, and treat stormwater runoff from small drainage areas by combining physical, chemical, and biological processes[3].	Established, dense turf with grass	Non-clumping form, stiff blades, dense coverage, and some tolerance of standing water
Infiltration swale	An infiltration swale is essentially a grass swale that has check dams placed along its length to create a flatter bed for temporarily holding stormwater.	Like a grass swale but with the addition of check dams to temporarily hold water	Incorporating a check dam in a swale improves surface storage capacity; temporary retention increases hydraulic-retention time, thereby enhancing infiltration and the potential for evapotranspiration.
Bioswale	A bioswale is a swale that incorporates a bioretention element, promoting infiltration and filtration primarily by employing engineered media with an underdrain[4,5].	Permeable soil mix or high flow engineered media; Underdrains may be present; Forebay or plunge pool is typical	A typical bioswale design includes (a) a forebay or plunge pool immediately upstream of the swale, (b) permeable soil mix or highly permeable engineered media, (c) geotextile fabric or layering of material to control flows within layers, and (d) a perforated underdrain pipe in a gravel drainage layer[5].
Wet swale	A wet swale functions similarly to stormwater wetlands and is designed to fit in linear environments.	Emergent wetland vegetation; Microtopographic pools and shallow areas; Seasonal high-water table; Wetland soils	A wet swale includes elements similar to a grass swale in addition to wetland soils, hydrology, and vegetation.

Table S2 Area of bioswales in each sub-catchment from 0.3% to 4% (Unit: m^2)

Sub-catchment	Bioswale (0.3%, baseline)	Bioswale (1 %)	Bioswale (2%)	Bioswale (3 %)	Bioswale (4 %)
1	114.41	1199.93	2399.85	3599.78	4799.71
2	638.96	357.97	715.94	1073.91	1431.88
3	500.51	293.97	587.94	881.92	1175.89
4	0	476.30	952.59	1428.89	1905.19
5	101.56	527.96	1055.93	1583.89	2111.86
6	463.46	268.94	537.88	806.81	1075.75
7	0	213.38	426.76	640.14	853.52
8	0	733.45	1466.89	2200.34	2933.78
9	210.64	428.35	856.69	1285.04	1713.38
10	253.02	272.29	544.59	816.88	1089.17
11	0	454.18	908.36	1362.54	1816.72
12	0	568.84	1137.69	1706.53	2275.38
13	161.55	420.74	841.47	1262.21	1682.95
14	0	740.05	1480.09	2220.14	2960.19
15	0	589.54	1179.09	1768.63	2358.18
16	0	509.70	1019.39	1529.09	2038.79
17	0	824.23	1648.45	2472.68	3296.90
18	204.02	157.66	315.33	472.99	630.66
19	0	279.36	558.73	838.09	1117.46
20	508.14	585.89	1171.77	1757.66	2343.54
21	546.90	568.29	1136.58	1704.87	2273.16
22	0	601.26	1202.53	1803.79	2405.05
23	0	534.09	1068.17	1602.26	2136.35
24	160.19	359.93	719.85	1079.78	1439.71
25	475.32	570.05	1140.09	1710.14	2280.18
26	0	21.70	43.40	65.10	86.80
27	0	22.07	44.15	66.22	88.29
28	0	23.73	47.45	71.18	94.90
29	0	16.57	33.14	49.71	66.28
30	0	45.90	91.81	137.71	183.62
31	0	39.61	79.23	118.84	158.46
32	256.70	217.87	435.75	653.62	871.49
33	0	404.95	809.89	1214.84	1619.78
34	0	608.63	1217.26	1825.89	2434.52
35	223.78	272.03	544.06	816.10	1088.13
36	0	555.28	1110.55	1665.83	2221.10
37	179.15	296.40	592.81	889.21	1185.62
38	0	1332.74	2665.47	3998.21	5330.94
Total	**4998.31**	**16393.82**	**32787.63**	**49181.45**	**65575.27**

Table S3 Modelling results of total runoff in each sub-catchment including baseline and climate scenarios

Sub-catchment	Baseline (Total runoff: mm)		SSP2-4.5(Total runoff: mm)		SSP3-7.0(Total runoff: mm)		SSP5-8.5(Total runoff: mm)	
	No SCP	SCP	No SCP	SCP	No SCP	SCP	No SCP	SCP
Sub-Catchment 1	159.86	133.60	242.49	214.76	240.34	212.67	252.89	224.77
Sub-Catchment 2	139.63	116.24	214.91	193.35	212.91	191.32	224.67	203.12
Sub-Catchment 3	75.85	58.87	131.68	122.52	130.08	120.78	139.65	131.00
Sub-Catchment 4	138.49	120.01	215.8	198.62	213.76	196.58	225.73	208.51
Sub-Catchment 5	140.38	120.47	218.3	199.39	216.25	197.34	228.27	209.28
Sub-Catchment 6	110.33	91.80	177.62	163.11	175.78	161.20	186.66	172.41
Sub-Catchment 7	191.63	173.39	283.8	264.48	281.46	262.16	295.01	275.47
Sub-Catchment 8	148.38	126.75	226.94	205.64	224.86	203.60	236.99	215.52
Sub-Catchment 9	135.68	116.53	210.03	192.95	208.04	190.93	219.7	202.68
Sub-Catchment 10	158.56	136.59	242.58	220.61	240.41	218.45	253.06	230.96
Sub-Catchment 11	156.33	137.39	237.86	219.34	235.73	217.21	248.17	229.56
Sub-Catchment 12	122.55	104.47	195.74	179.84	193.79	177.87	205.3	189.41
Sub-Catchment 13	156.05	135.64	238.08	217.96	235.94	215.83	248.41	228.20
Sub-Catchment 14	153.93	120.32	233.1	196.12	231.01	194.13	243.21	205.69
Sub-Catchment 15	118.69	101.97	189.2	175.47	187.29	173.52	198.53	184.94

Sub-Catchment 16	130.7	113.16	204.17	188.87	202.2	186.87	213.77	198.54
Sub-Catchment 17	131.36	111.19	206.52	187.68	204.52	185.69	216.26	197.32
Sub-Catchment 18	103.41	86.19	172.46	159.02	170.59	157.10	181.62	168.42
Sub-Catchment 19	125.55	107.67	199.79	183.92	197.81	181.93	209.43	193.58
Sub-Catchment 20	156.68	132.08	236.69	212.39	234.59	210.30	246.88	222.43
Sub-Catchment 21	160.41	135.14	243.43	217.83	241.26	215.69	253.86	228.06
Sub-Catchment 22	114.87	97.87	183.01	169.42	181.15	167.51	192.12	178.70
Sub-Catchment 23	204.53	174.82	300.31	265.64	297.9	263.36	311.83	276.44
Sub-Catchment 24	172.8	146.40	260.89	232.45	258.64	230.26	271.71	242.88
Sub-Catchment 25	159.33	134.19	243.02	217.30	240.85	215.16	253.48	227.54
Sub-Catchment 26	3131.27	2635.99	4968.23	4474.75	4919.4	4426.75	5201.17	4702.99
Sub-Catchment 27	574.2	499.54	1074.98	1011.61	1061.55	998.13	1139.27	1075.89
Sub-Catchment 28	796.38	693.35	1405.64	1316.59	1389.29	1300.08	1483.73	1395.26
Sub-Catchment 29	1637.69	1442.19	2675.24	2485.76	2648.02	2458.47	2805.22	2615.05
Sub-Catchment 30	32.88	34.12	86.53	88.18	85.01	86.67	94.24	95.85
Sub-Catchment 31	229.16	224.22	532.58	527.62	524.06	518.79	574.01	570.14
Sub-Catchment 32	152.11	129.45	234.58	212.19	232.44	210.06	244.91	222.42
Sub-Catchment 33	98.33	83.61	162.94	152.67	161.15	150.81	171.74	161.77

Sub-Catchment 34	138.93	120.27	214.97	197.81	212.95	195.78	224.8	207.62
Sub-Catchment 35	128.51	109.38	199.55	183.31	197.63	181.35	208.91	192.82
Sub-Catchment 36	124.87	107.46	196.9	182.04	194.96	180.07	206.37	191.60
Sub-Catchment 37	164	142.38	248.66	226.82	246.47	224.64	259.22	237.21
Sub-Catchment 38	282.83	217.72	395.87	317.82	393.06	315.32	409.02	329.51
Total runoff	11057.14	9472.43	17905.09	16375.85	17723.15	16194.38	18779.82	17243.56

Table S4 Modelling results of total nitrogen load in each conduit including baseline and climate scenarios

Conduits	Baseline (Nitrogen load: kg)		SSP2-4.5(Nitrogen load: kg)		SSP3-7.0(Nitrogen load: kg)		SSP5-8.5(Nitrogen load: kg)	
	No SCP	SCP	No SCP	SCP	No SCP	SCP	No SCP	SCP
Conduit_1	0.17	0.09	0.17	0.09	0.16	0.09	0.16	0.10
Conduit_2	0.17	0.16	0.17	0.13	0.17	0.12	0.17	0.13
Conduit_3	0.08	0.08	0.08	0.06	0.08	0.05	0.08	0.06
Conduit_4	18.47	13.47	21.37	17.55	21.38	17.50	21.56	17.85
Conduit_5	7.42	5.73	7.69	6.24	7.71	6.24	7.72	6.27
Conduit_6	0.14	0.09	0.14	0.09	0.14	0.09	0.14	0.09
Conduit_7	3.50	2.80	3.62	2.85	3.63	2.86	3.65	2.86
Conduit_8	3.44	2.52	3.61	3.04	3.62	3.04	3.64	3.06
Conduit_9	3.42	2.50	3.59	3.01	3.59	3.01	3.61	3.03
Conduit_10	3.40	2.48	3.57	2.99	3.58	2.98	3.60	3.01
Conduit_11	0.00	0.00	0.00	0.00	0.00	0.00	0.00	0.00
Conduit_12	7.00	5.62	9.09	8.05	9.06	8.02	9.23	8.24
Conduit_13	0.37	0.27	0.52	0.44	0.52	0.43	0.53	0.46
Conduit_14	0.00	0.00	0.00	0.00	0.00	0.00	0.00	0.00
Conduit_15	16.27	13.63	20.92	18.81	20.86	18.73	21.31	19.23

Conduit_16	17.91	14.53	23.95	20.92	23.86	20.81	24.53	21.48
Conduit_17	21.71	17.29	25.49	21.71	25.47	21.65	25.74	22.07
Conduit_18	0.15	0.09	0.31	0.12	0.31	0.12	0.34	0.12
Conduit_19	12.96	9.72	14.69	11.32	14.71	11.31	14.91	11.43
Conduit_20	10.32	7.85	13.01	9.92	13.01	9.88	13.31	10.13
Conduit_21	15.62	11.98	18.06	15.39	18.03	15.35	18.16	15.70
Conduit_22	17.34	12.41	19.66	14.37	19.69	14.36	19.86	14.54
Conduit_23	6.35	4.79	7.49	5.88	7.51	5.88	7.62	5.96
Conduit_24	5.99	4.94	8.16	6.32	8.11	6.28	8.46	6.56
Conduit_25	5.31	3.59	7.72	6.34	7.66	6.27	7.98	6.63
Conduit_26	6.63	5.36	8.57	7.63	8.55	7.60	8.71	7.79
Conduit_27	3.39	2.73	4.81	4.25	4.79	4.23	4.89	4.38
Conduit_28	10.34	8.24	13.59	11.51	13.55	11.45	13.90	11.83
Conduit_29	0.39	0.37	2.03	2.01	1.99	1.98	2.18	2.15
Conduit_30	0.22	0.15	2.91	2.61	2.82	2.53	3.30	3.00
Conduit_31	5.36	4.52	4.93	4.33	4.93	4.32	5.01	4.42
Conduit_32	14.36	11.90	16.00	14.14	15.98	14.11	16.20	14.36
Conduit_33	0.06	0.05	0.04	0.03	0.05	0.03	0.04	0.02

Conduit_34	2.51	1.87	2.12	1.98	2.13	1.99	2.12	1.93
Conduit_35	7.66	5.90	10.19	9.00	10.16	8.94	10.40	9.30
Conduit_36	17.39	14.26	20.53	18.14	20.50	18.07	20.81	18.49
Conduit_37	4.68	3.27	5.25	3.61	5.26	3.61	5.33	3.65
Conduit_38	1.95	1.45	2.04	1.61	2.04	1.61	2.05	1.62
Conduit_39	4.98	3.97	5.14	4.18	5.15	4.18	5.17	4.19
Conduit_40	11.83	6.19	11.74	6.93	11.80	6.96	11.60	7.03
Conduit_41	16.57	10.73	17.90	12.62	17.96	12.62	17.95	12.73
Conduit_42	2.32	1.66	2.49	1.88	2.50	1.88	2.50	1.88
Conduit_43	9.87	7.91	11.46	9.14	11.46	9.13	11.60	9.27
Conduit_44	0.94	0.56	1.65	1.15	1.64	1.14	1.75	1.21
Conduit_45	10.77	7.76	18.28	15.13	18.13	14.94	19.24	16.07
Conduit_46	4.94	2.46	5.63	2.72	5.66	2.72	5.68	2.76
Conduit_47	4.43	3.04	5.36	4.84	5.37	4.83	5.43	4.88
Conduit_48	6.30	4.58	8.47	7.19	8.44	7.13	8.65	7.43
Conduit_49	6.26	4.86	7.28	6.30	7.28	6.28	7.34	6.41
Conduit_50	9.57	8.22	10.15	8.94	10.15	8.93	10.21	8.99
Conduit_51	2.40	1.77	2.03	1.86	2.03	1.88	2.02	1.81

Conduit_52	0.38	0.28	0.53	0.44	0.53	0.44	0.53	0.46
Conduit_53	3.17	2.60	3.89	3.37	3.88	3.36	4.04	3.44
Conduit_54	5.73	4.82	7.16	6.07	7.14	6.04	7.32	6.20
Conduit_55	2.29	1.37	3.52	2.41	3.49	2.39	3.64	2.48
Conduit_56	1.62	1.02	3.86	2.85	3.80	2.81	4.11	3.06
Conduit_57	6.17	4.55	7.28	5.81	7.26	5.78	7.41	5.94
Conduit_58	11.18	9.15	16.62	14.75	16.52	14.63	17.16	15.32
Conduit_59	0.68	0.47	1.40	1.10	1.38	1.09	1.47	1.16
Conduit_60	0.00	0.00	0.03	0.02	0.03	0.02	0.04	0.03
Conduit_61	0.00	0.00	0.00	0.00	0.00	0.00	0.00	0.00
Conduit_62	0.00	0.00	0.00	0.00	0.00	0.00	0.00	0.00
Conduit_63	0.00	0.00	0.00	0.00	0.00	0.00	0.00	0.00
Conduit_64	0.00	0.00	0.00	0.00	0.00	0.00	0.00	0.00
Conduit_65	16.64	12.33	21.43	17.18	21.37	17.07	21.86	17.66
Conduit_66	5.26	3.93	5.53	4.62	5.54	4.62	5.57	4.66
Total nitrogen load	396.73	300.93	484.90	397.98	484.08	396.36	493.50	407.01

Table S5 Modelling results of total runoff in each sub-catchment considering the scale of bioswales

Sub-catchment	SSP2-4.5 (Total runoff: mm)				SSP3-7.0 (Total runoff: mm)				SSP5-8.5(Total runoff: mm)			
	Scale: 1%	Scale:2%	Scale:3%	Scale:4%	Scale: 1%	Scale:2%	Scale:3%	Scale:4%	Scale: 1%	Scale:2%	Scale:3%	Scale:4%
Sub-Catchment 1	208.72	201.31	192.90	184.00	206.63	199.22	190.84	181.96	218.72	211.24	202.78	193.75
Sub-Catchment 2	195.45	192.76	190.14	187.33	193.43	190.73	188.11	185.30	205.23	202.53	199.89	197.07
Sub-Catchment 3	123.94	121.88	119.69	117.37	122.21	120.14	117.95	115.62	132.41	130.38	128.21	125.92
Sub-Catchment 4	195.63	192.69	189.74	186.51	193.58	190.64	187.69	184.46	205.51	202.57	199.59	196.35
Sub-Catchment 5	196.86	193.60	186.49	186.49	194.81	191.54	184.44	184.44	206.76	203.46	196.34	196.34
Sub-Catchment 6	164.30	162.66	161.09	159.45	162.39	160.75	159.18	157.53	173.59	171.95	170.37	168.73
Sub-Catchment 7	261.72	259.13	256.59	254.04	259.40	256.81	254.26	251.72	272.71	270.12	267.57	264.98
Sub-Catchment 8	200.97	196.27	191.09	185.49	198.92	194.22	189.04	183.45	210.84	206.09	200.90	195.26
Sub-Catchment 9	191.64	189.06	186.46	183.69	189.63	187.04	184.45	181.67	201.37	198.77	196.15	193.36
Sub-Catchment 10	220.42	217.71	215.08	212.41	218.25	215.54	212.92	210.24	230.77	228.06	225.41	222.74
Sub-Catchment 11	216.27	213.28	210.22	207.00	214.14	211.14	208.08	204.87	226.49	223.49	220.39	217.17
Sub-Catchment 12	176.59	173.44	173.46	169.64	174.61	171.46	171.49	167.69	186.16	182.99	182.90	179.08
Sub-Catchment 13	216.12	213.13	210.11	206.92	213.98	210.99	207.97	204.78	226.36	223.36	220.30	217.11
Sub-Catchment 14	192.23	188.18	183.90	179.22	190.23	186.18	181.91	177.24	201.78	197.69	193.39	188.67
Sub-Catchment 15	172.86	170.21	167.43	164.36	170.91	168.26	165.48	162.41	182.32	179.64	176.86	173.76

Sub-Catchment 16	186.17	183.48	180.74	177.74	184.17	181.48	178.74	175.75	195.83	193.13	190.36	187.36
Sub-Catchment 17	183.27	178.74	173.61	168.03	181.27	176.75	171.62	166.05	192.90	188.34	183.19	177.57
Sub-Catchment 18	159.46	158.01	156.75	155.42	157.53	156.08	154.82	153.48	168.85	167.42	166.14	164.83
Sub-Catchment 19	181.20	178.77	176.37	173.78	179.20	176.77	174.36	171.78	190.87	188.43	186.04	183.46
Sub-Catchment 20	211.86	207.76	203.47	198.83	209.76	205.66	201.38	196.75	221.90	217.76	213.46	208.80
Sub-Catchment 21	217.68	213.58	209.32	204.69	215.54	211.44	207.18	202.56	227.90	223.76	219.49	214.85
Sub-Catchment 22	166.43	163.48	160.25	156.72	164.52	161.57	158.34	154.81	175.70	172.73	169.49	165.94
Sub-Catchment 23	258.26	251.31	243.95	236.16	255.98	249.02	241.67	233.88	269.05	262.05	254.68	246.86
Sub-Catchment 24	229.79	225.29	220.70	215.82	227.60	223.10	218.50	213.62	240.23	235.70	231.11	226.23
Sub-Catchment 25	216.62	212.45	208.06	203.31	214.48	210.30	205.92	201.17	226.85	222.64	218.26	213.48
Sub-Catchment 26	4412.98	4287.66	4144.34	3992.39	4365.10	4239.95	4097.02	3945.52	4640.35	4514.06	4369.49	4215.52
Sub-Catchment 27	1003.28	995.84	985.74	974.13	989.81	982.36	972.32	960.81	1067.38	1059.98	1049.54	1037.52
Sub-Catchment 28	1307.82	1295.78	1279.52	1260.71	1291.34	1279.31	1263.13	1244.42	1386.32	1374.18	1357.65	1338.27
Sub-Catchment 29	2469.34	2445.40	2413.20	2375.46	2442.09	2418.17	2386.04	2348.76	2598.35	2574.43	2541.72	2503.91
Sub-Catchment 30	87.59	87.23	86.86	86.53	86.07	85.70	85.32	84.99	95.27	94.94	94.59	94.28
Sub-Catchment 31	526.28	524.09	517.73	509.65	517.53	515.27	508.94	501.76	568.62	566.56	560.25	550.89
Sub-Catchment 32	212.66	210.03	207.59	205.07	210.54	207.91	205.46	202.94	222.90	220.27	217.80	215.28
Sub-Catchment 33	150.62	148.71	146.70	144.48	148.76	146.84	144.83	142.62	159.72	157.78	155.77	153.56

Sub-Catchment 34	194.56	191.27	187.84	184.08	192.53	189.23	185.81	182.05	204.36	201.03	197.59	193.81
Sub-Catchment 35	182.93	180.79	178.76	176.62	180.96	178.82	176.79	174.65	192.43	190.29	188.24	186.11
Sub-Catchment 36	179.23	176.43	173.50	170.28	177.26	174.45	171.53	168.31	188.79	185.95	183.02	179.78
Sub-Catchment 37	225.62	222.67	219.80	216.84	223.44	220.49	217.61	214.66	236.02	233.07	230.17	227.21
Sub-Catchment 38	303.42	288.36	272.02	255.05	300.93	285.88	269.58	252.66	315.03	299.84	283.38	266.22
Total runoff	16200.79	15912.44	15581.21	15225.71	16019.53	15731.21	15400.72	15047.38	17066.64	16776.68	16442.48	16082.03

Table S6 Modelling results of total nitrogen load in each conduit considering the scale of bioswales

Conduits	SSP2-4.5 (Nitrogen load: kg)				SSP3-7.0 (Nitrogen load: kg)				SSP5-8.5 (Nitrogen load: kg)			
	Scale: 1%	Scale:2%	Scale:3%	Scale:4%	Scale: 1%	Scale:2%	Scale:3%	Scale:4%	Scale: 1%	Scale:2%	Scale:3%	Scale:4%
Conduit_1	0.08	0.08	0.06	0.06	0.08	0.08	0.07	0.06	0.09	0.07	0.05	0.06
Conduit_2	0.12	0.12	0.10	0.10	0.13	0.11	0.11	0.09	0.13	0.10	0.10	0.09
Conduit_3	0.05	0.05	0.04	0.04	0.05	0.05	0.04	0.04	0.05	0.04	0.04	0.04
Conduit_4	16.79	16.14	15.60	15.01	16.73	16.06	15.52	14.94	17.08	16.44	15.90	15.34
Conduit_5	5.49	5.11	4.93	4.83	5.49	5.11	4.92	4.82	5.52	5.15	4.98	4.88
Conduit_6	0.04	0.04	0.03	0.03	0.04	0.04	0.03	0.03	0.04	0.04	0.03	0.02
Conduit_7	2.63	2.41	2.32	2.27	2.63	2.41	2.32	2.26	2.64	2.43	2.34	2.29
Conduit_8	2.94	2.79	2.71	2.64	2.94	2.78	2.70	2.64	2.96	2.82	2.73	2.68
Conduit_9	2.90	2.76	2.67	2.61	2.91	2.75	2.66	2.60	2.93	2.78	2.70	2.64
Conduit_10	2.89	2.74	2.65	2.58	2.89	2.73	2.64	2.59	2.91	2.76	2.68	2.62
Conduit_11	0.00	0.00	0.00	0.00	0.00	0.00	0.00	0.00	0.00	0.00	0.00	0.00
Conduit_12	7.99	7.84	7.67	7.44	7.95	7.80	7.63	7.40	8.17	8.02	7.86	7.63
Conduit_13	0.44	0.44	0.43	0.41	0.43	0.43	0.42	0.41	0.46	0.46	0.45	0.43
Conduit_14	0.00	0.00	0.00	0.00	0.00	0.00	0.00	0.00	0.00	0.00	0.00	0.00
Conduit_15	18.46	18.00	17.59	17.18	18.38	17.91	17.50	17.09	18.88	18.43	18.03	17.62

Conduit_16	20.61	20.06	19.53	19.06	20.50	19.94	19.41	18.94	21.18	20.62	20.11	19.63
Conduit_17	21.42	20.78	20.20	19.71	21.35	20.71	20.13	19.64	21.77	21.14	20.57	20.09
Conduit_18	0.10	0.09	0.10	0.10	0.10	0.09	0.10	0.10	0.11	0.12	0.10	0.10
Conduit_19	10.01	9.44	9.09	8.80	10.00	9.42	9.07	8.78	10.10	9.52	9.19	8.91
Conduit_20	8.83	8.44	8.16	7.95	8.80	8.40	8.13	7.91	9.02	8.63	8.35	8.13
Conduit_21	13.98	13.55	13.15	12.80	13.92	13.49	13.09	12.73	14.27	13.84	13.45	13.09
Conduit_22	12.86	12.19	11.86	11.64	12.84	12.17	11.84	11.62	13.01	12.32	12.00	11.79
Conduit_23	4.88	4.71	4.65	4.57	4.87	4.69	4.64	4.57	4.94	4.77	4.73	4.64
Conduit_24	5.26	4.96	4.71	4.52	5.22	4.93	4.68	4.50	5.50	5.16	4.89	4.70
Conduit_25	6.30	6.06	5.76	5.37	6.24	5.99	5.69	5.30	6.60	6.37	6.06	5.67
Conduit_26	7.56	7.43	7.27	7.08	7.53	7.39	7.24	7.04	7.72	7.59	7.44	7.24
Conduit_27	4.26	4.26	4.20	4.14	4.24	4.24	4.18	4.11	4.38	4.39	4.32	4.25
Conduit_28	10.95	10.67	10.43	10.20	10.88	10.61	10.36	10.14	11.26	10.98	10.73	10.51
Conduit_29	1.97	1.97	1.97	1.96	1.94	1.94	1.94	1.92	2.10	2.12	2.13	2.12
Conduit_30	2.57	2.54	2.40	2.32	2.49	2.46	2.32	2.24	2.95	2.91	2.78	2.69
Conduit_31	4.16	3.90	3.61	3.37	4.14	3.88	3.59	3.35	4.26	4.01	3.74	3.51
Conduit_32	13.67	13.08	12.89	12.51	13.63	13.04	12.85	12.46	13.91	13.31	13.14	12.76
Conduit_33	0.02	0.02	0.02	0.02	0.02	0.02	0.02	0.02	0.02	0.02	0.02	0.02

Conduit_34	1.87	1.69	1.63	1.57	1.88	1.71	1.64	1.58	1.82	1.65	1.59	1.53
Conduit_35	8.96	8.82	8.74	8.45	8.90	8.76	8.68	8.40	9.26	9.11	9.03	8.73
Conduit_36	17.68	17.11	16.84	16.39	17.62	17.04	16.77	16.32	18.04	17.46	17.20	16.75
Conduit_37	3.39	3.18	3.04	2.95	3.39	3.17	3.03	2.94	3.42	3.21	3.08	2.99
Conduit_38	1.51	1.42	1.37	1.34	1.51	1.42	1.36	1.33	1.52	1.44	1.38	1.35
Conduit_39	3.91	3.64	3.51	3.42	3.91	3.63	3.50	3.41	3.93	3.66	3.54	3.46
Conduit_40	4.16	3.83	3.72	3.65	4.16	3.83	3.72	3.65	4.20	3.86	3.74	3.68
Conduit_41	11.33	10.98	10.69	10.51	11.32	10.97	10.68	10.50	11.41	11.07	10.78	10.60
Conduit_42	1.63	1.59	1.54	1.48	1.63	1.59	1.54	1.49	1.64	1.60	1.55	1.49
Conduit_43	8.21	7.87	7.59	7.41	8.19	7.85	7.57	7.39	8.34	8.01	7.73	7.54
Conduit_44	1.14	1.11	1.06	1.02	1.12	1.09	1.05	1.01	1.20	1.17	1.12	1.07
Conduit_45	14.83	14.71	14.38	14.27	14.65	14.52	14.20	14.10	15.77	15.67	15.31	15.17
Conduit_46	2.25	2.01	1.91	1.88	2.25	2.01	1.91	1.88	2.27	2.03	1.93	1.90
Conduit_47	4.59	4.46	4.36	4.30	4.57	4.45	4.35	4.28	4.64	4.52	4.42	4.37
Conduit_48	7.16	7.04	6.86	6.70	7.11	6.99	6.82	6.65	7.38	7.25	7.07	6.90
Conduit_49	6.23	6.11	5.96	5.77	6.21	6.09	5.94	5.74	6.34	6.22	6.08	5.89
Conduit_50	8.52	8.09	7.93	7.77	8.51	8.09	7.92	7.75	8.58	8.15	8.00	7.84
Conduit_51	1.76	1.59	1.52	1.46	1.77	1.60	1.53	1.47	1.70	1.54	1.47	1.42

Conduit_52	0.44	0.44	0.43	0.42	0.44	0.44	0.43	0.41	0.46	0.46	0.46	0.44
Conduit_53	3.20	3.03	2.82	2.77	3.18	3.02	2.81	2.76	3.28	3.12	2.92	2.85
Conduit_54	5.82	5.54	5.24	4.96	5.80	5.51	5.21	4.92	5.96	5.68	5.40	5.13
Conduit_55	2.31	2.13	1.92	1.74	2.29	2.11	1.91	1.72	2.38	2.20	2.00	1.81
Conduit_56	2.83	2.76	2.64	2.52	2.78	2.71	2.60	2.48	3.03	2.96	2.85	2.72
Conduit_57	5.68	5.40	5.20	5.00	5.65	5.37	5.17	4.97	5.81	5.53	5.34	5.14
Conduit_58	14.49	14.29	14.05	13.81	14.37	14.17	13.93	13.68	15.05	14.87	14.63	14.38
Conduit_59	1.09	1.05	1.01	0.96	1.07	1.04	0.99	0.95	1.15	1.12	1.07	1.02
Conduit_60	0.02	0.02	0.02	0.02	0.02	0.02	0.02	0.02	0.03	0.03	0.02	0.02
Conduit_61	0.00	0.00	0.00	0.00	0.00	0.00	0.00	0.00	0.00	0.00	0.00	0.00
Conduit_62	0.00	0.00	0.00	0.00	0.00	0.00	0.00	0.00	0.00	0.00	0.00	0.00
Conduit_63	0.00	0.00	0.00	0.00	0.00	0.00	0.00	0.00	0.00	0.00	0.00	0.00
Conduit_64	0.00	0.00	0.00	0.00	0.00	0.00	0.00	0.00	0.00	0.00	0.00	0.00
Conduit_65	16.73	16.04	15.30	14.50	16.63	15.93	15.20	14.41	17.21	16.49	15.77	14.93
Conduit_66	4.46	4.23	4.10	4.01	4.46	4.23	4.09	4.00	4.49	4.27	4.15	4.06
Total nitrogen load	376.38	362.80	352.20	342.32	374.68	360.99	350.37	340.50	385.23	371.66	361.25	351.34

Table S7 Scale of low impact development practices in each sub-catchment[8,9]

Sub-catchment	Bioswale (m^2)	Rain Garden (m^2)	Permeable Pavement (m^2)	Total area (m^2)
1	114.41	8078.11	18298.46	26490.98
2	638.96	5732.68	5458.91	11830.55
3	500.51	4473.11	4482.97	9456.59
4	0	7544.05	7263.36	14807.41
5	101.56	7428.92	8051.28	15581.76
6	463.46	7268.17	4101.21	11832.84
7	0	2810.79	3253.98	6064.77
8	0	7154.73	11184.79	18339.52
9	210.64	7716.79	6532.13	14459.56
10	253.02	3980.58	4152.38	8385.98
11	0	6775.34	6926.10	13701.44
12	0	8350.87	8674.69	17025.56
13	161.55	6105.18	6416.10	12682.83
14	0	8901.10	11285.46	20186.56
15	0	11619.44	8990.34	20609.78
16	0	9354.27	7772.69	17126.96
17	0	9023.55	12569.15	21592.7
18	204.02	4295.96	2404.33	6904.31
19	0	4495.92	4260.20	8756.12
20	508.14	6276.15	8934.55	15718.84
21	546.90	5982.46	8666.21	15195.57
22	0	9881.32	9169.05	19050.37
23	0	2232.12	8144.63	10376.75
24	160.19	2755.38	5488.75	8404.32
25	475.32	5841.98	8693.00	15010.3
26	0	0	330.91	330.91
27	0	0	336.61	336.61
28	0	0	361.81	361.81
29	0	0	252.68	252.68
30	0	0	700.02	700.02
31	0	0	604.11	604.11
32	256.70	3191.01	3322.50	6770.21
33	0	9419.58	6175.28	15594.86
34	0	9183.29	9281.39	18464.68
35	223.78	5374.86	4148.40	9747.04
36	0	9842.71	8467.76	18310.47
37	179.15	3909.56	4520.05	8608.76
38	0	0	20323.75	20323.75
Total	4998.31	205000.00	250000.00	459998.31

Table S8 Basic data collection for SWMM in this study

Name	Description	Sources
Land use data	It refers to the proportions and spatial distribution of different types of land, including water bodies, non-commercial streets, commercial streets, buildings, and green space.	The data is primarily acquired through remote sensing techniques and sourced from the Ningbo (China) Tian Map. http://www.nbmap.gov.cn/
Drainage system data	The data encompasses pipelines, junctions, and the outfall.	The data is primarily based on the Chinese national standard document "GB50014-2021 Design Code for Outdoor Drainage", the SWMM User's Manual for Design and the Water Planning and Consulting Research Institute of the North China Municipal Engineering Corporation of China.
Rainfall data	Hourly rainfall data was measured at the Cicheng Rainfall Station for the entire day of September 14, 2022.	Zhejiang Province Water and Rain Monitoring and Warning Display Platform https://sqfb.zjsq.net.cn:8089/#/main/map/realtime-rain
Water quality data	This data set contains rainwater runoff characteristic values under different types of land use in Ningbo as well as pollutant accumulation parameter values.	This data refers to literature published in relevant Chinese journals.
Soil data	The soil type in the study area.	The soil data is from the Nanjing Institute of Soil Research, Chinese Academy of Sciences, and is scaled at 1:1000000.
Elevation data	A dataset that describes ground elevation information through regular grid points.	Local Government (Ningbo Planning and Geographic Information Center)

Table S9 Percentage of each type of land use in the study area

Land use Type	Description	Area (Ha)	Area proportion
Water bodies	All major river channels in the community	3.38	2.06
Non-commercial streets	Hardened surfaces near squares and major driveways in the community	32.34	19.73
Commercial streets	All sports venues in the community for physical exercise and roads in the business area	4.33	2.64
Buildings	All main buildings in the community	44.95	27.42
Green space	All vegetation coverage areas and permeable land within the community	78.94	48.15
	Total area	163.94	100

Table S10 Event Mean Concentration (EMC) value of stormwater runoff with Total Nitrogen (TN) and rainfall coefficient from different underlying surfaces

Land use type	EMC value of runoff with TN (Unit: mg/L)			Runoff coefficient
	Maximum value	Minimum value	Average or median	
Green space	1. 21	0. 18	0. 43	0.35
Water bodies	1.5	1.0	1.25	1.00
Commercial street	21. 05	2. 12	8. 71	0.55
Non-commercial street	14. 24	1. 49	4. 67	0.85
Rooftop	10. 61	1. 32	6. 08	0.85

(Notes: The EMC average value of runoff with TN in water bodies is 1.0 mg/l and the maximum value is 1.5 mg/l, which meets China's national surface water quality standard Class IV [6]. The EMC values of other different underlying surfaces were sourced from Xu, et al. [7] and were collected from field surveys during eight rainfall events from 2009 to 2019. The data with rainfall coefficient were sourced from "Guidelines for Sponge City Planning and Design of Ningbo City" issued by Ningbo Housing and Urban-Rural Development Bureau in 2019. This study used the middle value as its representative value when the value is not fixed and is within a range. Specifically, commercial streets refer to paved roads such as block stones, while non-commercial streets refer to concrete and asphalt pavements. The rooftops in this article include hard roofs, flat roofs without paving stones, and asphalt roofs.)

Table S11 Observed maximum rainfall from Yinzhou Rainfall station near this study area from 1983-2019

Year	Rainfall (mm)	Size Order	Empirical Frequency
1983	55.8	33	91.67
1984	77.2	14	38.89
1985	75	16	44.44
1986	57.8	32	88.89
1987	57.9	31	86.11
1988	74.8	18	50.00
1989	96.6	6	16.67
1990	77.5	13	36.11
1991	77.1	15	41.67
1992	98.5	4	11.11
1993	64.8	26	72.22
1994	71.9	21	58.33
1995	82.1	10	27.78
1996	60.9	29	80.56
1997	102.9	3	8.33
1998	103.7	2	5.56
1999	66	24	66.67
2001	65.1	25	69.44
2002	71.4	22	61.11
2004	80.8	11	30.56
2005	62.9	28	77.78
2006	58	30	83.33
2007	96.5	7	19.44
2008	53.3	34	94.44
2009	70	23	63.89
2010	50.9	35	97.22

2011	80	12	33.33
2012	88.1	8	22.22
2013	88	9	25.00
2014	98.4	5	13.89
2015	75	16	44.44
2016	73.3	20	55.56
2017	121.9	1	2.78
2018	74.1	19	52.78
2019	64.3	27	75.00

Notes: Observation rainfall are provided by the National Climate Center of the China Meteorological Administration from 1983 to 2019 (http://ncc-cma.net/cn/).

Table S12 Results based on the P-III Curve method and the same frequency amplification method

Type	Return period	Design Rainfall(mm/24h)	The magnification ratio during 24h rainfall (K)
Rainfall Event	100a	206.9	0.71
	50a	184.7	0.63
	20a	154.4	0.53
	10a	130.5	0.45
	5a	105.3	0.36
	2a	67.7	0.23

Table S13 Muifa rainfall and related rainfall in different return periods (Peak back)

Time	Muifa	Muifa-2a	Muifa-5a	Muifa-10a	Muifa-20a	Muifa-50a	Muifa-100a
00:00	0	0	0	0	0	0	0
01:00	0.3	0.069	0.108	0.135	0.159	0.189	0.213
02:00	0.7	0.161	0.252	0.315	0.371	0.441	0.497
03:00	1	0.23	0.36	0.45	0.53	0.63	0.71
04:00	1.3	0.299	0.468	0.585	0.689	0.819	0.923
05:00	0.6	0.138	0.216	0.27	0.318	0.378	0.426
06:00	1.6	0.368	0.576	0.72	0.848	1.008	1.136
07:00	0.1	0.023	0.036	0.045	0.053	0.063	0.071
08:00	0.4	0.092	0.144	0.18	0.212	0.252	0.284
09:00	2.7	0.621	0.972	1.215	1.431	1.701	1.917
10:00	2	0.46	0.72	0.9	1.06	1.26	1.42
11:00	4.8	1.104	1.728	2.16	2.544	3.024	3.408
12:00	10.3	2.369	3.708	4.635	5.459	6.489	7.313
13:00	20	4.6	7.2	9	10.6	12.6	14.2
14:00	18.6	4.278	6.696	8.37	9.858	11.718	13.206
15:00	24.2	5.566	8.712	10.89	12.826	15.246	17.182

16:00	10.7	2.461	3.852	4.815	5.671	6.741	7.597
17:00	17.4	4.002	6.264	7.83	9.222	10.962	12.354
18:00	23.2	5.336	8.352	10.44	12.296	14.616	16.472
19:00	34.9	8.027	12.564	15.705	18.497	21.987	24.779
20:00	39.7	9.131	14.292	17.865	21.041	25.011	28.187
21:00	41.7	9.591	15.012	18.765	22.101	26.271	29.607
22:00	23.4	5.382	8.424	10.53	12.402	14.742	16.614
23:00	6.9	1.587	2.484	3.105	3.657	4.347	4.899
24:00	6.1	1.403	2.196	2.745	3.233	3.843	4.331
Total Rainfall	292.6	67.298	105.336	131.67	155.078	184.338	207.746

Table S14 Related rainfall data in different return periods (Peak front)

Time	Muifa-2a	Muifa-5a	Muifa-10a	Muifa-20a	Muifa-50a	Muifa-100a
00:00	0	0	0	0	0	0
01:00	1.403	2.196	2.745	3.233	3.843	4.331
02:00	1.587	2.484	3.105	3.657	4.347	4.899
03:00	5.382	8.424	10.53	12.402	14.742	16.614
04:00	9.591	15.012	18.765	22.101	26.271	29.607
05:00	9.131	14.292	17.865	21.041	25.011	28.187
06:00	8.027	12.564	15.705	18.497	21.987	24.779
07:00	5.336	8.352	10.44	12.296	14.616	16.472
08:00	4.002	6.264	7.83	9.222	10.962	12.354
09:00	2.461	3.852	4.815	5.671	6.741	7.597
10:00	5.566	8.712	10.89	12.826	15.246	17.182
11:00	4.278	6.696	8.37	9.858	11.718	13.206
12:00	4.6	7.2	9	10.6	12.6	14.2
13:00	2.369	3.708	4.635	5.459	6.489	7.313
14:00	1.104	1.728	2.16	2.544	3.024	3.408
15:00	0.46	0.72	0.9	1.06	1.26	1.42

16:00	0.621	0.972	1.215	1.431	1.701	1.917
17:00	0.092	0.144	0.18	0.212	0.252	0.284
18:00	0.023	0.036	0.045	0.053	0.063	0.071
19:00	0.368	0.576	0.72	0.848	1.008	1.136
20:00	0.138	0.216	0.27	0.318	0.378	0.426
21:00	0.299	0.468	0.585	0.689	0.819	0.923
22:00	0.23	0.36	0.45	0.53	0.63	0.71
23:00	0.161	0.252	0.315	0.371	0.441	0.497
24:00	0.069	0.108	0.135	0.159	0.189	0.213
Total Rainfall	67.298	105.336	131.67	155.078	184.338	207.746

Table S15 Related rainfall data in different return periods (Peak middle)

Time	Muifa-2a	Muifa-5a	Muifa-10a	Muifa-20a	Muifa-50a	Muifa-100a
00:00	0	0	0	0	0	0
01:00	0.46	0.72	0.9	1.06	1.26	1.42
02:00	1.104	1.728	2.16	2.544	3.024	3.408
03:00	2.369	3.708	4.635	5.459	6.489	7.313
04:00	4.6	7.2	9	10.6	12.6	14.2
05:00	4.278	6.696	8.37	9.858	11.718	13.206
06:00	5.566	8.712	10.89	12.826	15.246	17.182
07:00	2.461	3.852	4.815	5.671	6.741	7.597
08:00	4.002	6.264	7.83	9.222	10.962	12.354
09:00	5.336	8.352	10.44	12.296	14.616	16.472
10:00	8.027	12.564	15.705	18.497	21.987	24.779
11:00	9.131	14.292	17.865	21.041	25.011	28.187
12:00	9.591	15.012	18.765	22.101	26.271	29.607
13:00	5.382	8.424	10.53	12.402	14.742	16.614
14:00	1.587	2.484	3.105	3.657	4.347	4.899
15:00	1.403	2.196	2.745	3.233	3.843	4.331

16:00	0.069	0.108	0.135	0.159	0.189	0.213
17:00	0.161	0.252	0.315	0.371	0.441	0.497
18:00	0.23	0.36	0.45	0.53	0.63	0.71
19:00	0.299	0.468	0.585	0.689	0.819	0.923
20:00	0.138	0.216	0.27	0.318	0.378	0.426
21:00	0.368	0.576	0.72	0.848	1.008	1.136
22:00	0.023	0.036	0.045	0.053	0.063	0.071
23:00	0.092	0.144	0.18	0.212	0.252	0.284
24:00	0.621	0.972	1.215	1.431	1.701	1.917
Total Rainfall	67.298	105.336	131.67	155.078	184.338	207.746

Table S16 Relevant parameters of low impact development practices (Source from Bi[8,9,] Nazari-Sharabian[10])

No.	Parameters	Bioswale	Rain Garden	Permeable Pavement
1	Berm Height (mm)	200	150	100
2	Vegetation Volume Fraction	0.1	0.1	0
3	Surface Roughness	0.2	0.2	0.015
4	Surface Slope	1	1	1
5	Soil_Thickness (mm)	500	500	200
6	Soil_Porosity	0.35	0.35	0.44
7	Soil_FieldCapacity	0.2	0.2	0.04
8	Soil_WiltingPoint	0.1	0.1	0.01
9	Soil_Conductivity (mm/h)	10	10	130
10	Soil_ConductivitySlope	28.8	28.8	45
11	Soil_SuctionHead	70.96	70.96	40
12	Storage_thickness (mm)	300	0	300
13	Storage_VoidRatio	0.5	0.75	0.75
14	Storage_SeepageRate (mm/h)	14.4	14.4	14
15	Storage_BlockageCoefficient	0	NA	0
16	Flow coefficient	2.1	NA	1.5
17	Flow Exponent	0.5	NA	0.5
18	Offset	6	NA	6
19	Pavement_Thickness(mm)	NA	NA	90
20	Pavement_VoidRatio	NA	NA	0.15
21	Pavement_Permeability (mm/h)	NA	NA	100
22	Pollutant Removals (%)	17.66	NA	32.24
23	Surface Width (m)	2.5	NA	25
24	Impervious Area Treated (%)	50	50	0
25	Pervious Area Treated (%)	50	50	0
26	Side Slope (run/rise)	NA	NA	NA

Table S17 Precipitation Percent Changes of rainfall intensities for SSP2-4.5, SSP3-7.0, and SSP5-8.5 scenarios in each month for 2020-2039 (Zhejiang Province, China)

Climate change scenario	Years 2020-2039		
	**Ensemble low (10th percentile)**	**Ensemble median (50th percentile)**	**Ensemble high (90th percentile)**
SSP2-4.5 (Jan)	-18.27%	1.38%	23.59%
SSP3-7.0 (Jan)	-21.55%	-4.23%	17.77%
SSP5-8.5 (Jan)	-16.72%	-1.24%	24.01%
SSP2-4.5 (Feb)	-19.43%	-0.52%	16.57%
SSP3-7.0 (Feb)	-12.76%	1.10%	22.03%
SSP5-8.5 (Feb)	-15.97%	2.69%	24.34%
SSP2-4.5 (Mar)	-18.82%	-0.06%	16.87%
SSP3-7.0 (Mar)	-13.70%	4.72%	19.24%
SSP5-8.5 (Mar)	-18.56%	2.82%	19.02%
SSP2-4.5 (Apr)	-17.28%	-3.43%	9.48%
SSP3-7.0 (Apr)	-16.17%	-2.27%	12.24%
SSP5-8.5 (Apr)	-15.62%	-2.02%	10.37%
SSP2-4.5 (May)	-10.32%	4.60%	21.58%
SSP3-7.0 (May)	-12.59%	-0.37%	13.43%
SSP5-8.5 (May)	-10.00%	3.77%	18.63%
SSP2-4.5 (Jun)	-11.28%	4.20%	18.64%
SSP3-7.0 (Jun)	-11.76%	1.90%	19.89%
SSP5-8.5 (Jun)	-10.89%	6.17%	23.97%
SSP2-4.5 (Jul)	-12.93%	3%	18.39%
SSP3-7.0 (Jul)	-18.30%	-0.62%	13.37%
SSP5-8.5 (Jul)	-20.28%	-1.29%	16.84%
SSP2-4.5 (Aug)	-11.62%	2.82%	22.48%
SSP3-7.0 (Aug)	-15.91%	0.52%	22.06%
SSP5-8.5 (Aug)	-10.35%	5.68%	23.98%
SSP2-4.5 (Sep)	-23.01%	17.66%	39.58%
SSP3-7.0 (Sep)	-20.08%	12.74%	38.63%
SSP5-8.5 (Sep)	-16.88%	14.27%	44.23%
SSP2-4.5 (Oct)	-31.38%	-7.76%	22.86%
SSP3-7.0 (Oct)	-30.20%	-0.23%	31.63%
SSP5-8.5 (Oct)	-40.78%	-4.72%	35.82%
SSP2-4.5 (Nov)	-37.90%	-7.88%	17.04%
SSP3-7.0 (Nov)	-33.53%	-7.47%	24.12%
SSP5-8.5 (Nov)	-35.34%	-0.57%	27.70%
SSP2-4.5 (Dec)	-25.49%	-2.47%	23.56%
SSP3-7.0 (Dec)	-22.34%	-3.87%	22.18%
SSP5-8.5 (Dec)	-25.07%	1.61%	32.80%

Notes: source from climate change knowledge portal of the World Bank Group for Zhejiang Province, China (https://climateknowledgeportal.worldbank.org/country/china/climate-data-projections). Based on the relevant data from the period: 1995-2014 with Multi-Model Ensemble.

Table S18 Maximum number changes of consecutive dry days for SSP2-4.5, SSP3-7.0, and SSP5-8.5 scenarios in September for 2020-2039 (Zhejiang Province, China)

Climate change scenario	Years 2020-2039（unit: days）		
	Ensemble low (10th percentile)	Ensemble median (50th percentile)	Ensemble high (90th percentile)
SSP2-4.5	-4.04	-0.7	2.51
SSP3-7.0	-4.25	-0.5	3.4
SSP5-8.5	-4.16	-0.54	2.96

Notes: source from climate change knowledge portal of the World Bank Group for Zhejiang Province, China (https://climateknowledgeportal.worldbank.org/country/china/climate-data-projections). Based on the relevant data from the period: 1995-2014 with Multi-Model Ensemble.

Table S19 Calculation results of runoff coefficient in the study area

Land use type	$F_i\varphi_i$	F_i	Runoff coefficient
Water bodies	3.38	3.38	1
Commercial street	2.38	4.33	0.55
Non-commercial street	27.49	32.34	0.85
Rooftop	38.21	44.95	0.85
Green space	11.84	78.94	0.15
Calculated results	83.30	163.94	**0.51**

Table S20 Performance of low impact development practices in Cicheng New Town[8]

Elements	Total area (ha)	Removal Rate of TN (2019, March 5th)	Removal Rate of TN (2019, March 28th)	Removal Rate of TN (2019, May 15th)	Removal Average Rate of TN
Rain garden	11.4	26.02%	23.66%	22.76%	24.15%
Permeable Pavement	12.5				
Bioswale	0.5				

Note: The width of the constructed bioswale area in Cicheng New Town is 2.5m, the total length is 1995.9 m which is determined by the length of the road where it is located.

Table S21 Model validation process based on total nitrogen removal rate in terms of water quality (Rainfall on 14, Sept 2022)

Uncertainty Parameters	Range	Initial values	Adjusted values											
TN rate constant	0-1	0.1	0.1	0.1	0.1	0.1	0.5	0.25	0.24	0.2	0.16	0.17	0.168	0.169
Power/Sat.Constant	0-1	0.1	0.1	0.1	0.1	0.1	0.5	0.25	0.24	0.2	0.16	0.17	0.168	0.169
Green-Space coefficient (Washoff)	0.18-1.21	0.18	0.36	0.72	1.44	1.08	1.08	1.08	1.08	1.08	1.08	1.08	1.08	1.08
Water-bodies coefficient (Washoff)	1.0-1.5	1	1.1	1.2	1.3	1.2	1.2	1.2	1.2	1.2	1.2	1.2	1.2	1.2
Commercial-Street coefficient (Washoff)	2.12-21.05	2.12	4.24	8.46	16.92	12.69	12.69	12.69	12.69	12.69	12.69	12.69	12.69	12.69
Non-commercial-Street coefficient (Washoff)	1.49-14.24	1.49	2.98	5.96	11.92	8.94	8.94	8.94	8.94	8.94	8.94	8.94	8.94	8.94
Roof-Top coefficient (Washoff)	1.32-10.61	1.32	2.34	4.68	9.36	7.02	7.02	7.02	7.02	7.02	7.02	7.02	7.02	7.02
Removal rate (%)	24.15 (Validation value)	19.26	20.14	20.94	20.92	20.97	31.41	25.94	25.78	25.13	23.75	24.19	24.11	24.147

Table S22 Model validation process based on comprehensive coefficient in terms of water quantity (Rainfall on 14, Sept 2022)

**Uncertainty Parameters**	**Range**	**Initial values**	**Adjusted values**							
River-Roughness	0.030-0.070	0.030	0.05	0.05	0.05	0.05	0.05	0.05	0.05	0.05
Con-Roughness	0.011-0.026	0.011	0.015	0.015	0.015	0.015	0.015	0.015	0.015	0.015
N-Imperv	0.011-0.024	0.011	0.018	0.024	0.024	0.024	0.024	0.021	0.021	0.021
N-Perv	0.15-0.8	0.15	0.475	0.8	0.8	0.8	0.64	0.64	0.64	0.64
Dstore-Imperv (mm)	0.05-5	1.27	2.525	5	5	5	3.76	3.76	3.76	3.76
Dstore-Perv (mm)	0.05-10	2.54	5.025	10	10	10	7.51	7.51	7.51	7.51
Zero-Imperv (%)	0-100	0	50	80	80	80	65	65	65	65
Suction Head	49.02-320.04	290.07	290.07	290.07	184.53	116.78	116.78	256.78	256.78	256.78
Conductivity	0.254-120.40	0.51	0.51	0.51	5.1	62.75	62.75	31.37	15.66	23.52
Initial Deficit	0.097-0.375	0.11	0.11	0.11	0.236	0.306	0.306	0.271	0.271	0.271
Modelling comprehensive coefficient	0.51 (reference value)	0.9380	0.9271	0.9139	0.8779	0.5075	0.5069	0.5069	0.5524	0.5108

Notes: The range of adjusted uncertainty parameters is sourced from the SWMM manual

Figure S1 Key components and treatment processes of green swales and four types of green swales including (a) grass swales, (b) infiltration swales, (c) bioswales and (d) wet swales (source: Lingwen Lu[2])

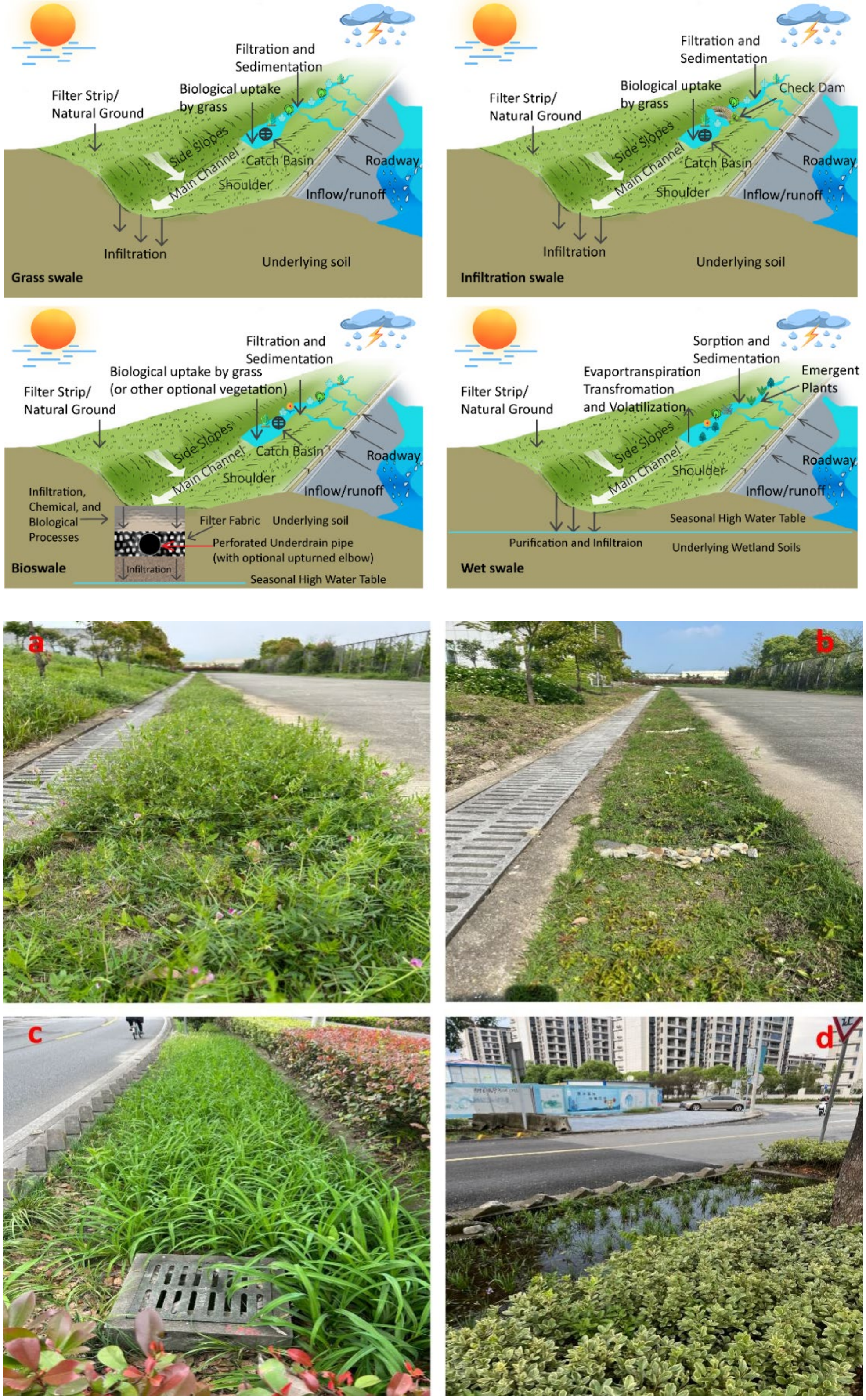

Figure S2 P III frequency curve fitting diagram (rainfall event during 24 hours) based on data from Yinzhou Rainfall Station

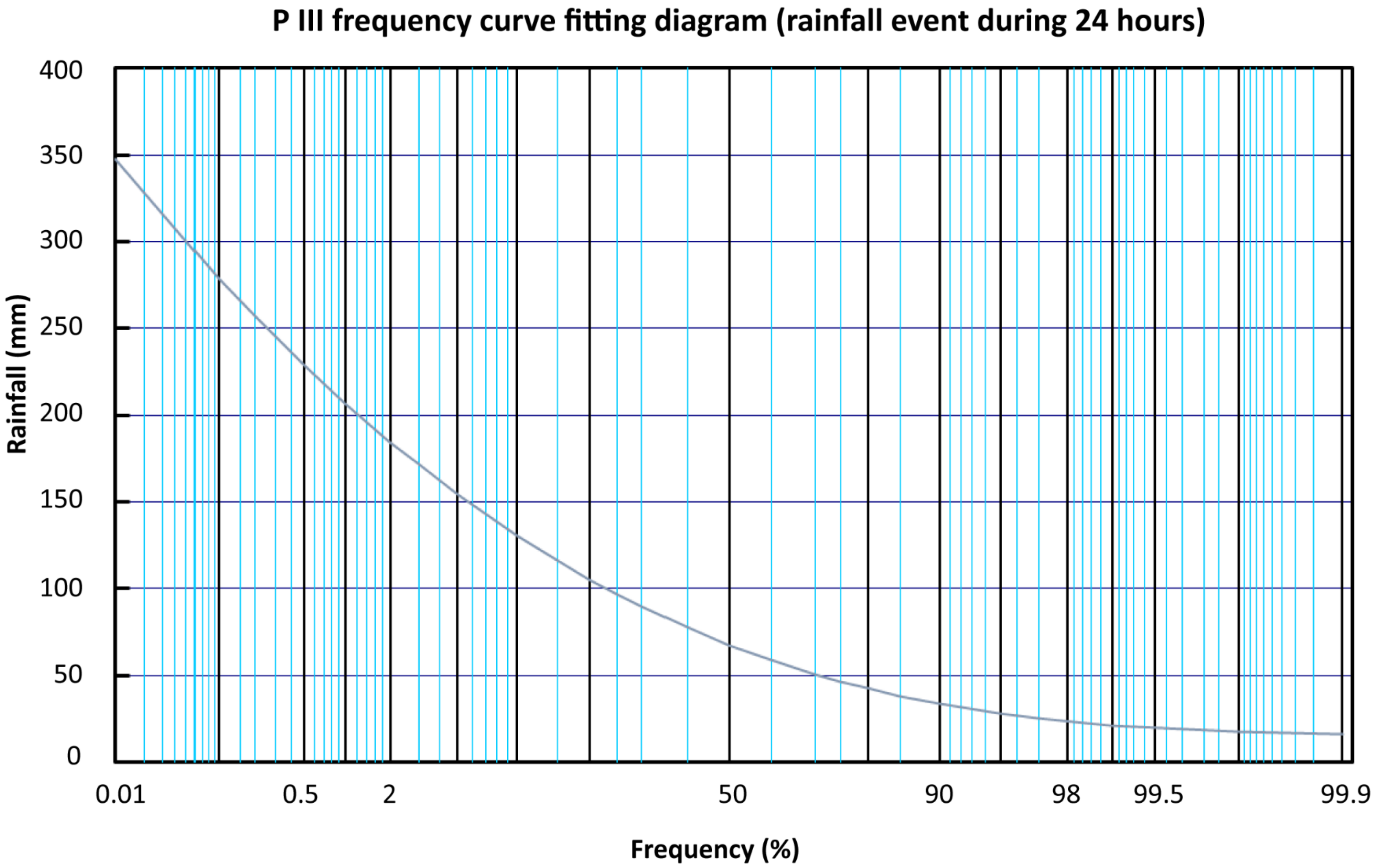

Figure S3 The baseline bioswale in the Cicheng New Town (the area of bioswale is 0.3% of the catchment)

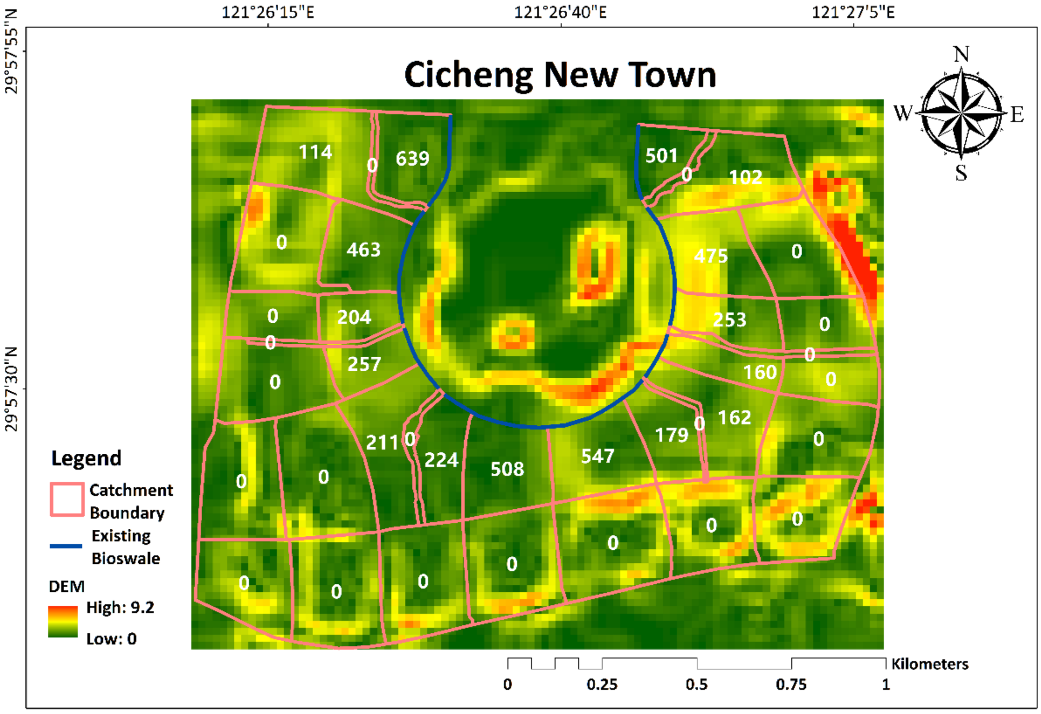

Figure S4 Increased bioswale in the Cicheng New Town (the area of bioswale is 2% of the catchment)

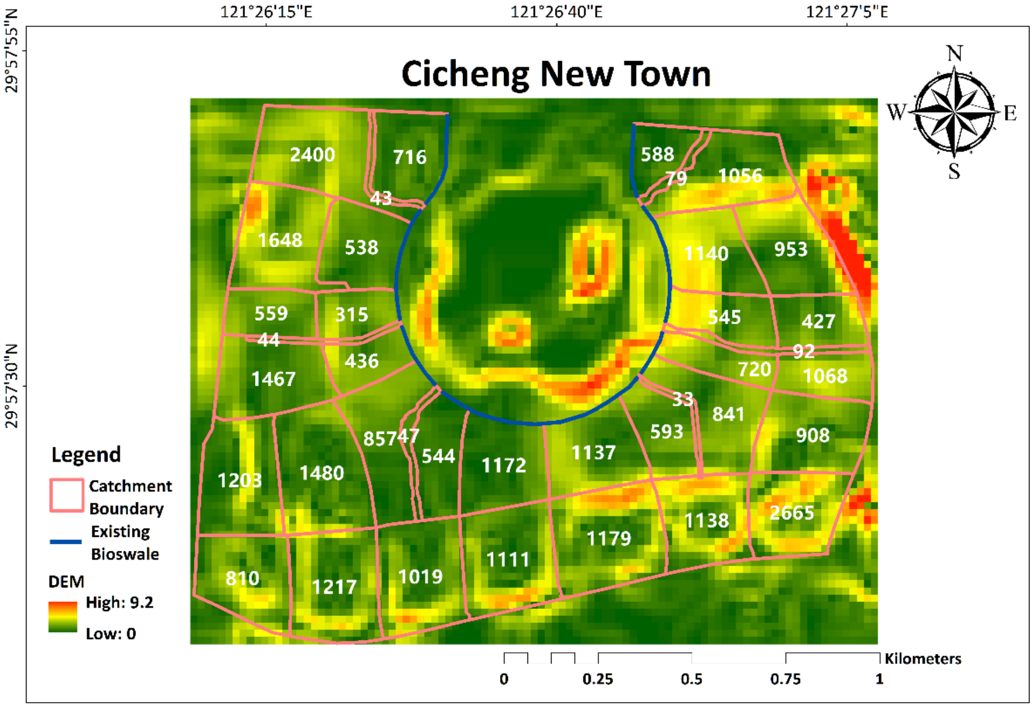

Figure S5 Increased bioswale in the Cicheng New Town (the area of bioswale is 4% of the catchment)

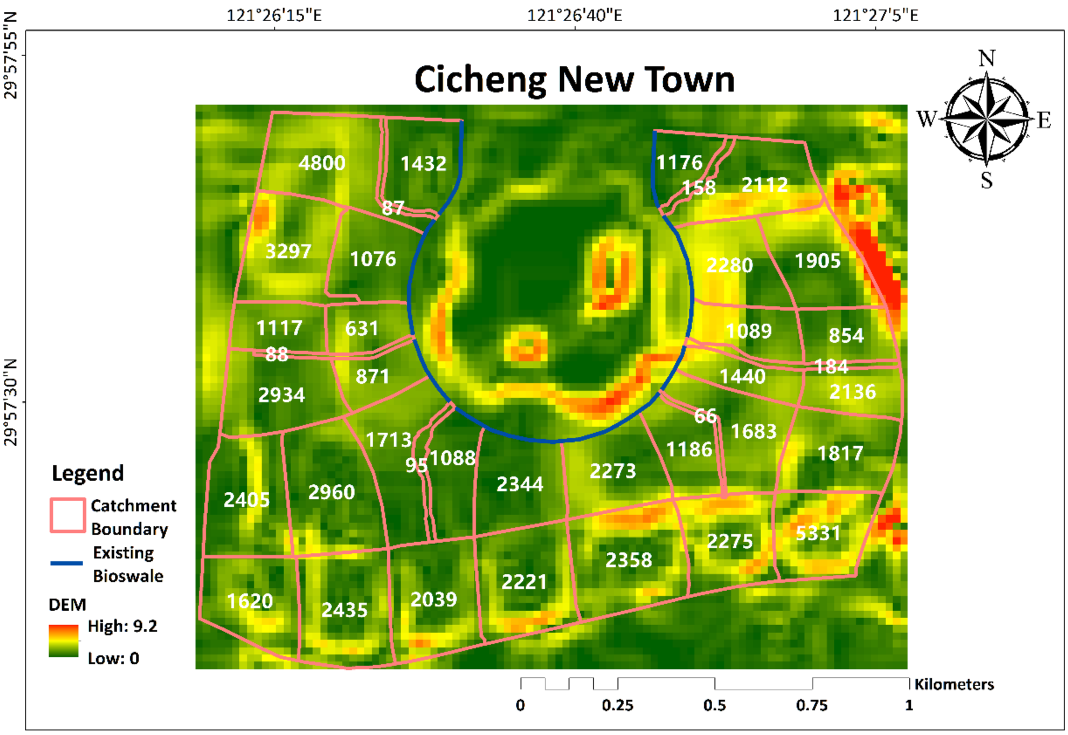

References

1. Ekka S A, H. W. F. Swale Terminology for Urban Stormwater Treatment. Urban Waterway Series, NC State University Cooperative Extension, Raleigh, North Carolina. https://content.ces.ncsu.edu/swale-terminology-for-urban-stormwater-treatment (2020).
2. Lu, L., Chan, F. K. S., Johnson, M., Zhu, F. & Xu, Y. The development of roadside green swales in the Chinese Sponge City Program: Challenges and opportunities. *Frontiers of Engineering Management*, https://doi.org/10.1007/s42524-023-0267-z (2023).
3. Barrett, M. E., Walsh, P. M., Malina, J. F. & Charbeneau, R. J. Performance of vegetative controls for treating highway runoff. *Journal of Environmental Engineering-Asce* **124**, 1121-1128, https://doi.org/10.1061/(ASCE)0733-9372(1998)124:11(1121) (1998).
4. R. D. Christianson, B. J. B., J. C. Hayes, K. Gasem, and G. O. Brown. "Modeling Effectiveness of Bioretention Cells for Control of Stormwater Quantity and Quality" in *Modeling Effectiveness of Bioretention Cells for Control of Stormwater Quantity and Quality*, https://doi.org/10.1061/40737(2004)37. (World Water and Environmental Resources Congress. Salt Lake City, UT., 2004).
5. Purvis, R. A. *et al.* Evaluating the Water Quality Benefits of a Bioswale in Brunswick County, North Carolina (NC), USA. *Water* **10**, 16, https://doi.org/10.3390/w10020134 (2018).
6. Xu, L. & Chen, X. Analysis of sponge transformation project of Ecotope in Cicheng New Town, Nin gbo, China (*In Chinese*). *Chinese Horticulture Abstracts* **6**, 88-90 https://kns.cnki.net/kcms2/article/abstract?v=zcLOVLBHd2x1VEiXtXnNYgIB-O3Ck8nghJzRIOE_Tr4ncd0eLqI-gRVVYnTNsoLkrxzKEIpKZnjxT5TjbALR5yDm-I9wWN0SINnGMnWwY_W46EPk6RqaVUeGpfxtnI2xxmsQrQWzd6s3GIKLyYrFUg==&uniplatform=NZKPT&language=CHS (2018).
7. Xu, Y.-j. *et al.* Analysis of rainwater runoff pollution characteristics of various typical underlying sur faces in Ningbo. *Environmental Science in Chinese* **41**, 3275-3284, https://kns.cnki.net/kcms2/article/abstract?v=zcLOVLBHd2zhl4PhQnGMy8OSd6LuAeFdqhYFDxlHYRFJU-HkKR-LSvpcO35aytrHxOwU49lvBvZDdTrmm2FnAOPPip0VBxgRacKnGQG6hL4y6EN6sePb-bzk4bXdxYzmJnC9jMWFbFr2lP3vVoFylQ==&uniplatform=NZKPT&language=CHS (2020).
8. Bi, J. Simulation and analysis of Nitrogen pollution control of surface runoff in sponge city based on SWMM. *Ningbo University. Master Thesis. (in Chinese)* https://kns.cnki.net/kcms2/article/abstract?v=zcLOVLBHd2xU-2hW_zMMT47oyndoRia8CUUIcVFq0Vw9whRu6Oad8S7_2DPWlfEpnvDpFnYOVKJp22lt6alm2esLA4NuUck0gt4h2D0MZP4uvhiQkSgn7-VLmphTL_q5ZHmU1bhCFyUWwbEtFt3QLQ==&uniplatform=NZKPT&language=CHS (2020).
9. Yan Zaitian & Wenshen, D. Water-sensitive urban design of Cicheng New Town. *Urban and rural c onstruction (in Chinese)* **7**, 11-14 https://kns.cnki.net/kcms2/article/abstract?v=zcLOVLBHd2wWxJdJtA1wSJPTGvRFCpTkLMna4LkwSCMnhOqI_dZ2EeexEg0QoOgqmQd1GlVfuJouX8AmrMxX72DYRPG52GqX9vbriVFuwk34ijqk7yI2iMtePDWFCzsaPar6ZZLlc4ZfpPVkRT0oDA==&uniplatform=NZKPT&language=CHS (2017).
10. Nazari-Sharabian, M., Taheriyoun, M. & Karakouzian, M. Surface runoff and pollutant load response to urbanization, climate variability, and low impact developments - a case study. *Water Sci. Technol.-Water Supply* **19**, 2410-2421, https://doi.org/10.2166/ws.2019.123 (2019).
